# Ultrafast photoinduced band splitting and carrier dynamics in chiral tellurium nanosheets

**DOI:** 10.1038/s41467-020-17766-5

**Published:** 2020-08-10

**Authors:** Giriraj Jnawali, Yuan Xiang, Samuel M. Linser, Iraj Abbasian Shojaei, Ruoxing Wang, Gang Qiu, Chao Lian, Bryan M. Wong, Wenzhuo Wu, Peide D. Ye, Yongsheng Leng, Howard E. Jackson, Leigh M. Smith

**Affiliations:** 1 Department of Physics and Astronomy, University of Cincinnati, Cincinnati, OH 45221 USA; 2 Department of Mechanical & Aerospace Engineering, The George Washington University, Washington, D.C. 20052 USA; 3 School of Industrial Engineering, Purdue University, West Lafayette, IN 47907 USA; 4 School of Electrical and Computer Engineering, Purdue University, West Lafayette, IN 47907 USA; 5 Department of Chemical & Environmental Engineering, Materials Science & Engineering Program, University of California, Riverside, Riverside, CA 92521 USA

**Keywords:** Electronic properties and materials, Semiconductors, Topological insulators, Ultrafast photonics

## Abstract

Trigonal tellurium (Te) is a chiral semiconductor that lacks both mirror and inversion symmetries, resulting in complex band structures with Weyl crossings and unique spin textures. Detailed time-resolved polarized reflectance spectroscopy is used to investigate its band structure and carrier dynamics. The polarized transient spectra reveal optical transitions between the uppermost spin-split *H*_4_ and *H*_5_ and the degenerate *H*_6_ valence bands (VB) and the lowest degenerate *H*_6_ conduction band (CB) as well as a higher energy transition at the L-point. Surprisingly, the degeneracy of the *H*_6_ CB (a proposed Weyl node) is lifted and the spin-split VB gap is reduced upon photoexcitation before relaxing to equilibrium as the carriers decay. Using ab initio density functional theory (DFT) calculations, we conclude that the dynamic band structure is caused by a photoinduced shear strain in the Te film that breaks the screw symmetry of the crystal. The band-edge anisotropy is also reflected in the hot carrier decay rate, which is a factor of two slower along the *c*-axis than perpendicular to it. The majority of photoexcited carriers near the band-edge are seen to recombine within 30 ps while higher lying transitions observed near 1.2 eV appear to have substantially longer lifetimes, potentially due to contributions of intervalley processes in the recombination rate. These new findings shed light on the strong correlation between photoinduced carriers and electronic structure in anisotropic crystals, which opens a potential pathway for designing novel Te-based devices that take advantage of the topological structures as well as strong spin-related properties.

## Abstract (web summary)

The complex band structure and carrier decay response upon photoexcitation of the chiral semiconductor tellurium remain to be unveiled. Here, the authors report unusual dynamic band modifications in Te near band-edge structure due to photoinduced symmetry breaking and strong anisotropy in carrier decay dynamics.

## Introduction

The isolation of graphene^1^ has stimulated extensive research in van-der Waals (vdWs) materials, revealing new physics, as well as offering building blocks for novel electronic devices^2–4^. Beyond graphene and the transition metal dichalcogenides (TMDs), a variety of exotic materials such as topological insulators (TIs)^5^ and Weyl semimetals (WSMs)^6^ have been recently discovered, which display unique electronic structure and chiral spin texture on the Fermi surface. Most of these materials involve heavy elements such as Bi, Sb, Te, Se, etc., suggesting that strong spin-orbit interaction (SOI) is the key to the complex electronic structure in these materials. Elemental tellurium (Te) has strong interest due to its chiral nature and unique spin texture and topological features in the band structure^7–11^. Both 2D Te nanostructures and high mobility field-effect devices have been demonstrated^12^.

**Fig. 1:**
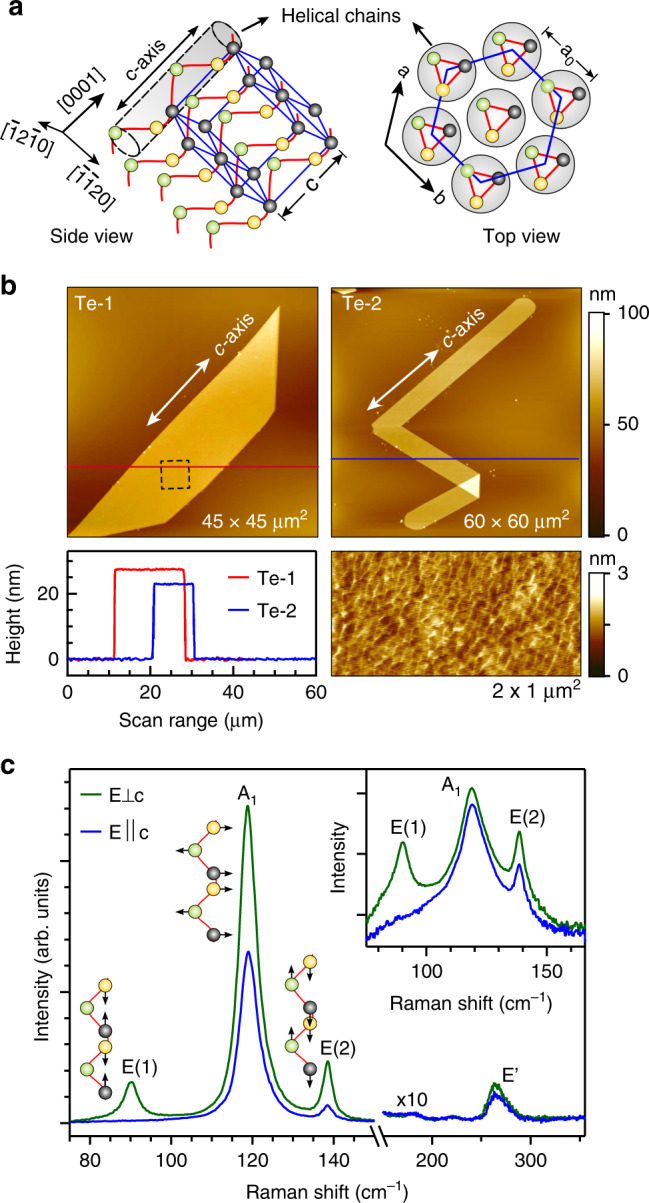
Structure and morphology of Te nanosheets. **a** Crystal structure of trigonal Te (left image) in which spheres of different colors represent Te atoms within the helical chains (shaded region) arranged in a hexagonal array along the growth direction, i.e., the *c*-axis. Pattern with blue lines shows a hexagonal unit cell along [0001]. The right image is the top (surface) view of the crystal from the [0001] direction, showing ab–plane (basal plane) and surface lattice parameter (*α*_0_). **b** AFM topographies of the Te nanosheets used for optical measurements (symbolized by Te-1 and Te-2) and their cross section profiles (red and blue lines). Close-up height scan on Te-1 (dotted rectangle) is shown on bottom right, which exhibits a relatively smooth surface with an average corrugation of <2 nm. **c** Representative Raman spectra acquired on these samples using a 633-nm laser excitation source with two perpendicularly polarized configurations with respect to the *c*-axis, i.e., $E||c$ and $E \bot c$. Three prominent optical modes, as indicated by *A*_1_, *E*(1), and *E*(2) modes, respectively, are detected with $E \bot \,c$ while only *A*_1_ and *E*(2) modes are clearly visible with $E||c$. Inset shows a log scale plotting of the data, showing that the *E*(1) mode is symmetry forbidden for $E||c$. A weak second order harmonic of *E*-mode is seen in the spectra and indicated by the symbol *E*′.

Trigonal Te is a 1D vdWs crystal where 3-fold helices of covalently bonded Te atoms are weakly coupled in a hexagonal close-packed array^13,14^. Each helix is parallel to the *c*-axis (Fig. 1a), and so crystals display either right-handed (space group: $P3_121 - D_3^4$) or left-handed (space group: $P3_221 - D_3^6$) symmetries^15^. Gyrotropic properties have been observed in Te such as a strong optical rotatory power^16^, the circular photogalvanic effect^17,18^, current-induced magnetization^19^, etc. This makes trigonal Te a unique material for polarization optics, multiferroics, and spintronics. The lack of mirror and inversion symmetries result in a unique radial spin texture in the Te band structure^7–9^, in contrast to Rashba systems^20^ or topological materials^21^. The spin degeneracy of the uppermost valence bands (VBs) at the H- or H′-points in the Brillouin zone is lifted in momentum space due to the SOI and the breaking of inversion symmetry while the lowest conduction band (CB) is doubly spin-degenerate and protected by the 3-fold screw symmetry of the helices^7–10^. Trigonal Te is insulating at ambient pressure but transforms to a metallic phase under hydrostatic pressure^22,23^. The band structure of Te exhibits a topological electronic structure with a number of Weyl nodes near the H-point, particularly the spin-degenerate *H*_6_ CB minimum^9,10^. The topological phase transition from a trivial semiconductor to a Weyl semimetal (WSM) is predicted under applied external pressure when the spin-polarized uppermost VBs and CB are inverted across the band gap^8,24^. Since a strong piezoelectric effect has been reported in bulk and nanostructured Te^25–27^, strain can also be generated in response to an applied electric field and vice versa. The response of the electronic structure to laser excitation could enable manipulation and control of topological phases in Te. The unique chiral electronic structure offers the ability to control the electronic charge and spin degrees of freedom in the absence of a magnetic field for applications in spintronics.

While ground-state optical absorption with light polarized parallel or perpendicular to the *c*-axis has been carried out^15,28–32^, very little is known about carrier dynamics or higher lying states. The ground-state band-edge absorption anisotropy is explained by the symmetry properties of the Te crystal^32,33^. However, at higher energies the Te band structure exhibits a large number of multi-valley nested degenerate band structures that results in high thermoelectric efficiencies^34^. Probing carrier dynamics in such a wide energy region provides a unique opportunity to investigate inter- and intra-valley carrier decay processes in addition to the fundamental recombination lifetime^35^.

Polarized optical transitions are studied in a Te nanosheet at 10 K and 300 K by exciting with a 1.51 eV pump pulse and probing with a tunable mid-infrared probe pulse polarized parallel and perpendicular to the *c*-axis. Polarized optical transitions and carrier dynamics at the band-edge and higher lying states are probed over nanoseconds and analyzed with the support of numerical calculations. Transient anisotropic band modifications observed in the measurements are proposed to be caused by a photoinduced piezoelectric shear strain generated in the Te film and explicitly calculated by ab initio density functional theory (DFT) band structure calculations. Energy and polarization-dependent kinetics are evaluated to determine the dominant relaxation channels of photoexcited carriers in Te.

## Results

### Sample characteristics

The Te nanostructures are chemically synthesized and dispersed onto a Si/SiO_2_ substrate (see Methods section)^12^. The covalently bonded Te chains are parallel to the *c*-axis of the hexagonal crystal while the basal ab–plane is perpendicular to the chain (Fig. 1a). The *c*-axis lies in the nanosheet plane, enabling polarized optical measurements. Figure 1b shows AFM images of the nanostructures and their orientation with respect to the *c*-axis. The thickness varies between 20 nm and 30 nm (Fig. 1b). The surface morphology displays an atomically smooth surface, without noticeable oxidation.

The sample orientations were determined by polarized micro-Raman spectroscopy (see Methods) with the laser either polarized parallel ($E||c$) or perpendicular ($E \bot c$) to the *c*-axis. With $E \bot c$, we observe three sharp Raman peaks at 119 cm^−1^, 90 cm^−1^, and 138.5 cm^−1^ corresponding to the *A*_1_ (symmetric intra-chain breathing in the basal plane), and the doubly degenerate *E*(1) (rigid chain rotation in the basal plane), and *E*(2) (asymmetric stretching along the chain or the *c*-axis) modes, as indicated in Fig. 1c^36^. The weak second order harmonic *E*′ at 266 ± 1 cm^−1^ is also seen. Logarithmic plots of the data (see inset of Fig. 1c) confirm that the *E*(1) mode is symmetry forbidden for $E||c$.

### Polarized transient reflectance spectroscopy

**Fig. 2:**
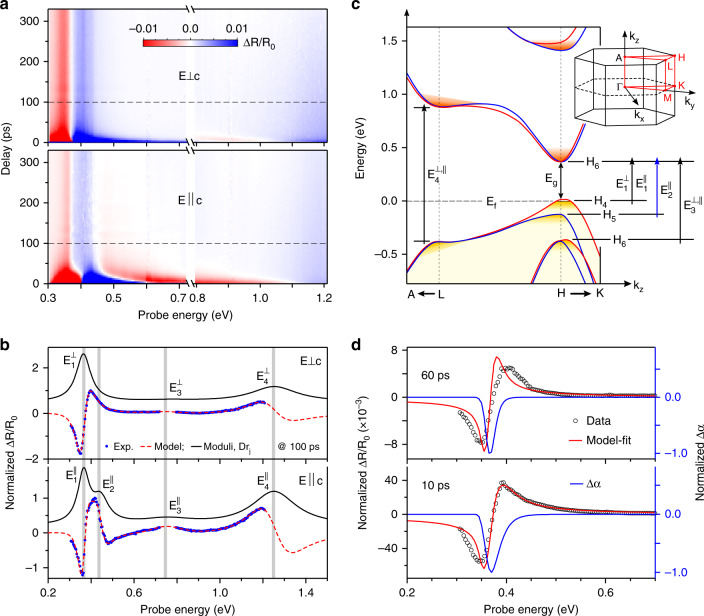
Polarized spectral map and modeling of the TR response of a Te nanosheet. **a** Two-dimensional false color maps of the pump-probe delay dependent polarized TR spectra over extended probe energies, showing strong features with distinctive dynamics at different energies. **b** TR spectra (blue circles) acquired at a 100 ps time delay with two orthogonal probe polarizations. Each spectrum is fitted with a simple model described in the main text (red dashed lines). Calculated moduli Δ*ρ*_*j*_ of each fit are also plotted with a vertical offset (black lines). Both the model fits and the moduli show transient features corresponding to the series of optical transitions in the Te bulk band structure. **c** Schematic band structure of bulk Te and possible optical transitions around the high symmetry H and L points of the Brillouin zone near the band-edge region. Observed transition energies in the TR spectra at different band-edge regions are indicated. On the top right, the first Brillouin zone of Te and various high symmetry points are indicated. **d** Transient spectra (black open circles) around the fundamental gap region measured with the $E \bot c$ configuration of the probe pulse at two different time delays. Each spectrum is well reproduced using a band-filling model described in the main text (red lines). The calculated change in absorption Δ*α*, normalized by the peak values for clarity, at respective delay times are also superimposed with scales on the right (blue lines). Overall, the model captures the main features of the spectra and extracts important band parameters such as band gap and Fermi energy.

Polarized transient reflectance (TR) measurements are performed using pump-probe methods (see Methods section and Supplementary Note 2). The change in the reflectance of the polarized tunable probe beam with the 1.51 eV pump pulse present, ${\mathrm{{\Delta}}}R( {E,t} ) = ( {R_{{\mathrm{pump}} - {\mathrm{on}}} - R_{{\mathrm{pump}} - {\mathrm{off}}}} )$, is measured as a function of both energy (*E*) and delay (*t*). The Δ*R*(*E*, *t*) data are normalized by the polarized probe reflectance *R*_0_(*E*) (pump off) at all probe energies *E*, and recorded as Δ*R*/*R*_0_. Figure 2a displays a false color map of the TR spectra, Δ*R*/*R*_0_ measured over 0.3–1.2 eV energies for the probe polarized parallel ($E||c$) and perpendicular ($E \bot c$) to the *c*-axis. The spectra show derivative-like anti-symmetric features in both the low-energy (~0.3–0.5 eV) and high-energy (~1–1.2 eV) regions and a relatively weaker and broader signal near ~0.6–0.8 eV, indicating a series of interband optical transitions. The $E||c$ spectra exhibit an additional anti-symmetric feature near ~0.45 eV, which is not present for $E \bot c$, suggesting polarization-sensitive transient optical response in Te samples. We first quantify each of these features and compare to band-to-band optical transitions from band-structure calculations.

### Ground-state optical transitions

The TR signal near the band-edge decays almost completely within first 30 ps (Fig. 2a) with a weak residual signal persisting over a longer time. In Fig. 2b we display normalized spectra at 100 ps delay for each polarization. The derivative-like features of the transient signal at low energies (0.34–0.45 eV) exhibit a clear difference for the two polarizations while high-energy features (0.9–1.2 eV) are nearly identical. These features persist over long time scales, suggesting their origin is due to weak perturbation of the dielectric response caused by photoexcitation of electrons and holes. The energies of optical transitions are accurately determined by using a derivative Lorentzian lineshape^37^,

1 $$\frac{{{\mathrm{{\Delta}}}R}}{{R_0}}\left( E \right) \simeq \mathop {\sum }\limits_{j = 1}^n Re\left[ {A_je^{i\varphi _j}\left( {E - E_j + i{\mathrm{{\Gamma}}}_j} \right)^{ - 2}} \right],$$

where *n* represents the number of interband transitions involved, *E* is the photon energy of the probe beam, and *A*_*j*_, *φ*_*j*_, *E*_*j*_, and Γ_*j*_ are the amplitude, phase, transition energy, and the energy broadening parameter of the *j*th feature, respectively. Such a form cannot provide physical insights, but provides an efficient way of accurately determining the transition energies. The best-fit lineshapes to spectra are shown with dashed-lines in Fig. 2b. The spectra with $E \bot c$ can be fitted with three resonances (*n* = 3) while the spectra with $E||c$ can be fitted with four resonances (*n* = 4). Moduli (absorption profiles) of individual resonances obtained from Eq. (1), are shown with minor vertical shifts for clarity. The values of the transition energies are indicated by vertical dotted lines.

The transition energies determined above are compared with the detailed band structure around the H and H′ high symmetry points of the bulk Brillouin zone of Te^7,9,38^, (see Fig. 2c and inset). The five bands closest to the Fermi level probed in the TR experiment include three successive VBs and one CB minimum. The uppermost two VBs (*H*_4_ and *H*_5_) are spin-polarized (non-degenerate) while the lower lying VB (*H*_6_ or $H_6^{VB}$) and the lowest CB (*H*_6_ or $H_6^{CB}$) are Rashba-like 2-fold spin-degenerate and protected by the screw symmetry of the helices^7–9^. The $H_6^{CB}$ CB minimum has been proposed to be a Weyl node crossing^9^. The intrinsic large SOI causes a camelback feature in the *H*_4_ VB along the *H*-*K* direction, showing a weakly indirect band gap in Te. In our experiments, the 1.51 eV pump photon energy generates hot electrons and holes, and these carriers scatter to band minima through inter- and intra-band processes. The time-delayed mid-IR probe pulse interrogates the carrier occupation of each band by monitoring the change in the reflectivity at specific interband transition energies (see vertical arrows). The energies of these three optical transitions nearly match the energy of each band as predicted by theory (Fig. 2c).

The lowest $H_4 \to H_6^{CB}$ transition with $E \bot c$ at 100 ps is $E_1^ \bot = 0.363 \pm 0.002\,{\mathrm{eV}}$, which is almost identical with the transition with $E||c$, $E_1^{||} = 0.366 \pm 0.002\,{\mathrm{eV}}$. Both transitions appear to be direct and are nearly identical in energy, though a one-third weaker response is observed with $E||c$, caused by the higher reflectivity *R*_0_ of the sample with $E||c$ as compared to $E \bot c$^28^. We do not see any evidence of a forbidden or indirect transition with $E||c$ as discussed in previous work^15,28^. The transition $E_1^ \bot$ is ~30 meV larger than the previous optical absorption results of the bulk band gap of Te^15,28,39^. The higher absorption-edge is caused by the Moss-Burstein shift of the Fermi energy below the maximum at *H*_4_ in our naturally *p*-doped samples (Fig. 2c). Hall measurements^12^ in similar nanosheets have indicated a free hole density of ~5 × 10^18^ cm^−3^. The optical transition $H_5 \to H_6^{CB}$ is observable only with $E||c$, consistent with the expected dipole allowed transition between these two states^40^. The transition energy at longer delays is $E_2^{||} = 0.448 \pm 0.005\,{\mathrm{eV}}$, so we conclude that the energy separation between the Fermi level in the *H*_4_ VB and the *H*_5_ VB is $E_2^{||} - E_1^ \bot = 0.085\, {\mathrm{{eV}}}$. We determine a 30 meV Fermi energy within the *H*_4_ band, indicating the gap between the *H*_4_ and *H*_5_ band-edges to be ${\mathrm{{\Delta}}}E = 0.115\,{\mathrm{eV}}$, which coincides with the 11 micron wavelength hole absorption band, previously measured in a semi-insulating bulk single crystal of Te^29,41^.

The energy and polarization dependence of the optical transitions is strongly consistent with band structure calculations. In addition to the direct transitions at the band-edge, a higher energy transition near $E_3^{||, \bot } = 0.75 \pm 0.005\,{\mathrm{eV}}$ is observed which is weakly sensitive to probe polarization. The one order of magnitude weaker TR signal at this energy suggests that this transition might be indirect in **k**-space. The valence band $H_6^{VB}$ (Fig. 2c) is doubly spin-degenerate with one spin-state showing a maximum outside ${\mathbf{k}}_{\mathbf{z}} = 0$ at the H-point before extending to the Weyl node^7,9^. This indirect transition is likely between $H_6^{VB}$ and $H_6^{CB}$. The transition at $E_3^{||, \bot }$ falls right in the gap of our photon source, 0.72–08 eV, which adds a small uncertainty in estimating the transition energy. A higher lying transition at $E_4^{||, \bot }\sim 1.25 \pm 0.005\,{\mathrm{eV}}$, is nearly four-times the gap. The TR response of this transition is a factor of two larger than the $E_3^{||, \bot }$ response but much weaker than the band-edge transitions. Due to the probe pulse energy range, only one-half of the derivative-like line shape is accessible, causing uncertainties in extracting the precise energy and polarization anisotropy of this transition. This higher energy transition cannot result from higher lying valence or conduction bands at the H-point. Based on the band-structure of Te near high symmetry points in the Brillouin zone^7,9^, we find only one vertical transition possible at the L-point that matches with $E_4^{||, \bot }$.

### Estimation of band-edge parameters

A band-filling analysis is used to model the TR spectra at the band-edge. Our samples are highly p-type^12^, and so the lowest observed optical transition is between the Fermi level below the VB edge to the bottom of the CB. The TR lineshapes result from changes to the complex index of refraction caused by the photoexcited electrons and holes. The additional holes cause a slight downshift of the Fermi energy at the *H*_4_ VB, which changes the absorption onset for optical transitions between the *H*_4_ VB hole gas to the $H_6^{CB}$ CB. Using Kramers-Kronig analysis, the calculated change in the absorption coefficient (Supplementary Note 5) is transformed to a change in the real part of the index of refraction. The free parameters in this calculation are the density of the doped hole gas in *H*_4_ band, the density and temperature of the photoexcited carriers (electron-hole pairs), and the fundamental gap energy *E*_*g*1_($H_4 \to H_6^{CB}$). The lineshapes are calculated assuming parabolic electron and hole bands with effective masses 0.6 *m*_e_ and 0.4 *m*_e_, respectively^7^. The calculated Fermi energy is smaller because of the substantial valley degeneracies of *H*_4_ and $H_6^{CB}$ bands, 12 and 24, respectively. Figure 2d shows TR spectra (black open circles) and model fits (red lines) near the band-edge measured with $E \bot c$ at two different delay times. The blue lines overlapped on each spectrum with scales on the right show a decrease in the absorption (photoinduced bleaching) due to the pump excitation. Best fits show a doped hole density of *N*_*d*_ ~ 2 × 10^18^ cm^−3^, with a Fermi energy of *E*_f_ ~ 30 meV, and a fundamental gap between *H*_4_ and $H_6^{CB}$ of $E_g\sim 0.32 \pm 0.005\,{\mathrm{eV}}$. Our estimated doping agrees well with those obtained by Hall measurements on similar nanosheets^12,42^. The photoexcited carrier density at the band-edge reaches to Δ*N*_eh_ ~ 1 × 10^18^ cm^−3^ at a delay time of 10 ps. The zero crossing of the lineshape occurs at the minimum of the absorption change Δ*α* (right scale in Fig. 2d) at 0.368 eV, which matches closely with $E_1^ \bot$ and provides a consistency check of the Aspnes form fit (Eq. (1)). The estimated fundamental gap is consistent with previous low temperature optical measurements on moderately doped bulk samples^15,28,39^. The band parameters obtained from the model fittings are tabulated in Supplementary Tables 1 and 2.

### Dynamic evolution of optical transitions

**Fig. 3:**
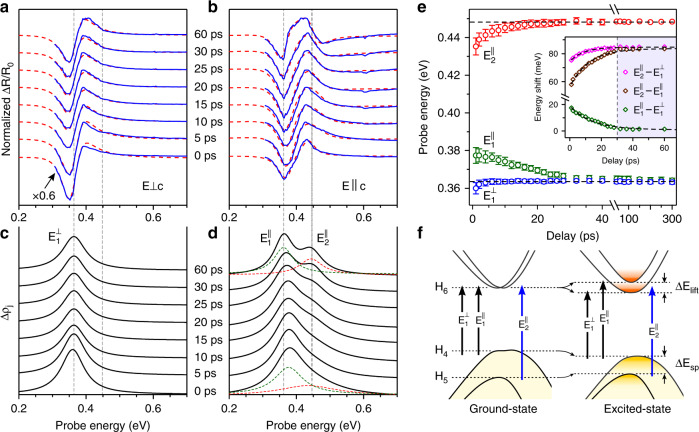
Transient response of polarized optical transitions in Te nanosheet. **a**, **b** A series of selected TR spectra (blue lines) recorded at different pump-probe delay times for both $E \bot c$ and $E||c$ polarizations. Dashed red lines are model fits of each corresponding spectrum. **c**, **d** Moduli of each corresponding fit for both polarizations. Vertical dashed gray lines are guides to the eye to indicate the systematic shift of peak position. **e** Transition energies $E_1^ \bot$, $E_1^{||}$, and $E_2^{||}$ extracted from the fits and plotted as a function of delay for both polarizations. Error bars are estimated from the least square fitting procedure. While the $E_1^ \bot$ remains mostly stable over a long delay, the $E_1^{||}$, and $E_2^{||}$ undergo gradual blue and redshifts as delay progresses before they remain steady after ~30 ps. The inset shows the time evolution of anisotropic spectral shifts, as quantified by $E_1^{||} - E_1^ \bot$ and $E_2^{||} - E_1^ \bot$ for fundamental and higher energy band-gap anisotropy and $E_2^{||} - E_1^{||}$ for the gap between uppermost VBs excluding the doping induced Fermi energy shift. Superimposed dashed lines on each curve are exponential fits for a guide to the eye. The shaded region is shown to indicate the static region. **f** Ground-state and photoinduced band structure (note exaggerated energy scales) around the H-point near the band gap, showing photoinduced lifting ${\mathrm{{\Delta}}}E_{lift}$ of the $H_6^{CB}$ band and narrowing of the gap ${\mathrm{{\Delta}}}E_{sp}$ between the uppermost VBs. The involved optical transitions are indicated by vertical arrows.

**Fig. 4:**
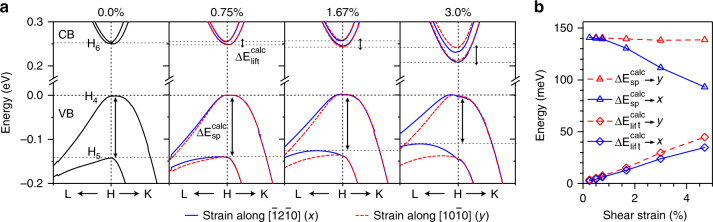
Te band structure under shear strain. **a** Evolution of Te band structures around the H-point near the Fermi level with increasing shear strain. Blue lines correspond to the shear strain along $\left[ {\bar 12\bar 10} \right](x)$ while red dashed lines correspond to the strain along $[10\bar 10](y)$ directions, which is applied on the two (0001) planes of the Te crystal. **b** Variations of spin degeneracy lifting ${\mathrm{{\Delta}}}E_{lift}^{calc}$ of the *H*_6_ CB (or $H_6^{CB}$) and the spin-split gap ${\mathrm{{\Delta}}}E_{sp}^{calc}$ between frontier *H*_4_ and *H*_5_ VBs as a function of the magnitude of the shear strain (written on the top in percentage) along both directions, as indicated by *x* and *y*, respectively.

Figure 3a, b shows selected TR spectra at different delay times for orthogonal polarizations near the band-edge. Apart from amplitude, the lineshape does not change for $E \bot c$, likely caused by the relatively weak pump excitation. The spectra with $E||c$, however, display changes caused by a band shift. Due to the complex Te VB structure, accurate modeling of the $E||c$ spectra is not trivial; by assuming minimal broadening, we analyze these spectra by fitting with Eq. (1). Aspnes et al.^43^ have shown that increasing the broadening by a factor four or amplitude by a factor of 100 has minimal impact (<3 meV) on the determination of the transition energy. As described earlier, the spectra with $E \bot c$ fit well with a single transition energy (*n* = 1) while the spectra with $E||c$ fit well with two transition energies (*n* = 2), close to each other but phase-shifted by 180° (dashed red lines). Corresponding moduli (absorption bands) of the spectra obtained from the fits are shown in Fig. 3c, d. The time evolution of the peak position exhibits a shift in the transition energies for both polarizations and are plotted as a function of delay in Fig. 3e. The lowest transition energy with $E \bot c$ remains steady at the ground-state value of $E_1^ \bot = 0.36\,{\mathrm{eV}}$ except a minor down-shift (~3 meV) within 2 ps. Such an abrupt down-shift of band gap is attributed to band-gap renormalization, which results from many-body effects arising from the hot electron-hole plasma^44^. The lowest energy transition with $E||c$, however, is shifted by ~20 meV higher than $E_1^ \bot$ near time zero. The higher energy $E_2^{||}$ transition between $H_5 \to H_6^{CB}$ with $E||c$ (dipole forbidden with $E \bot c$), is red-shifted by ~10 meV near time zero. Interestingly, both of these transitions with $E||c$ gradually relax back to their respective late time energies within ~30 ps. The transient recovery is shown by plotting the delay dependence of the energy difference near the first and second band-edge regions, i.e., $E_1^{||} - E_1^ \bot$ and $E_2^{||} - E_1^ \bot$, respectively, as well as spin-split VB gap, i.e., $E_2^{||} - E_1^{||}$ (inset of Fig. 4e). The maximum shift $E_1^{||} - E_1^ \bot$ near time zero is ~20 meV while the $E_2^{||} - E_1^{||}$ is reduced by ~30 meV. An exact evaluation of the spin-split gap is hindered by the lack of well-resolved transitions from *H*_5_ to the closely spaced $H_6^{CB}$ lifted bands. The strong line broadening of the $E_2^{||}$ TR spectra as compared to the $E_1^{||}$ transition at early times can be seen in the green and red dashed fits in Fig. 3d. The shifts in both transitions gradually return to steady states as the majority of photoexcited carriers relax to the ground-state within first ~30 ps.

The physical origin of this dynamic shift in the first 30 ps is complex. Because the shifts are both positive and negative for different transitions, it is not possible to explain the shifts due to ultrafast processes including excitonic, many body, or band filling effects. Excitonic interactions are ruled out due to the very low exciton binding energy ($E_b\sim 0.5\,{\mathrm{meV}}\; < < \;E_g$) in bulk Te^45^. An ultrafast band-gap collapse upon high density (>1% of valence electrons or ~10^21^ cm^−3^) excitation is predicted in Te^46^, which is not possible here (*n*_eh_ ~ 10^18^ cm^−3^). The only remaining possibility is a transient change in the lattice structure of Te causing a dynamic shift in the band-edges. Recent theoretical studies have predicted strain-induced band modifications in a Te crystal^8,24^. Under uniaxial or hydrostatic pressure, all CBs and VBs edges move vertically, either decreasing (compressive strain) or increasing (tensile strain) the gap, but the degeneracy of the $H_6^{CB}$ band is protected. Shear strain, while affecting all the bands, also lifts the $H_6^{CB}$ band degeneracy by breaking the screw symmetry of the Te helix^8^. We therefore rule out uniaxial or hydrostatic stress effects because the gap or $E_1^ \bot$ transition with $E \bot c$ remains unchanged upon photoexcitation. Moreover, there is no change in the band-edge shift for the pump pulse aligned parallel or perpendicular to *c*-axis, in contrast to the uniaxial strain effect in anisotropic Te^8,47^. Thus shear strain must contribute dominantly in our experiment. Furthermore, an induced shear strain should change the spin-split VB gap due a change in SOI^48^; this is observed in our experiment. We propose that these dynamic shifts in the first 30 ps arise due to lifting of the $H_6^{CB}$ spin degeneracy and the decrease of the spin-split VB gap by photoinduced shear strain generation in Te helices, as shown schematically in Fig. 3f. The lifting of the $H_6^{CB}$ band degeneracy ${\mathrm{{\Delta}}}E_{lift}$ increases the energy of the $E_1^{||}$ and decreases the $E_2^{||}$ transitions as well as the spin-split VB gap Δ*E*_*sp*_ consistent with experiments. These strain-dependent shifts observed in $E||c$ spectra relax within the first ~30 ps, suggesting strong correlation between the density of photoexcited carriers and electronic structure.

To support our proposed strain-induced band modifications, we have used density functional theory (DFT) to calculate the Te band structure under different strains (see Methods section). Under zero or uniaxial/hydrostatic strains, the band structure shows identical features as described in both early^8^ and recent^24^ studies (Supplementary Note 1). As expected, VB and CB edges move vertically but preserve the band degeneracy of $H_6^{CB}$. Shear strains applied along $\left[ {\bar 12\bar 10} \right](x)$ or $[10\bar 10](y)$ directions, on the two (0001) surfaces, however, cause the 2-fold degeneracy of $H_6^{CB}$ to lift even for a small 0.75% shear strain, forming two nested energy valleys (Fig. 4a). Furthermore, both the splitting ${\mathrm{{\Delta}}}E_{{{lift}}}^{{{calc}}}$ of the $H_6^{CB}$ band increases, and the spin-split VB gap ${\mathrm{{\Delta}}}E_{{\mathrm{sp}}}^{{\mathrm{calc}}}$ decreases systematically with strain (Fig. 4a, b). The fundamental gap is only weakly affected by shear strain. The calculated degeneracy lifting ${\mathrm{{\Delta}}}E_{{{lift}}}^{{{calc}}}$ of the $H_6^{CB}$ under 3% strain along the *x*-direction is 24 meV, and along the *y*-direction is 30 meV. Both values are higher than our experimentally estimated value of ${\mathrm{{\Delta}}}E_{lift}\sim 17\,{\mathrm{meV}}$ at *t* = 0 ps. The calculated spin-split VB gap ${\mathrm{{\Delta}}}E_{sp}^{calc}$ is hardly affected by *y*-direction strain but noticeably decreases for *x*-direction strain. This suggests that the $[\bar 12\bar 10](x)$ shear strain is dominant. These shear strain calculations capture all the features of the dynamic band modifications we observe in our measurements. However, the magnitude of ~2–3% strain required to match our measurements is somewhat large. Considering the elastic strain field in the Te flake induced by laser excitation is a complex three-dimensional strain field (the calculations assume uniform shear strain), we anticipate that if the equivalent von Mises shear strain induces the same shear lifting of $H_6^{CB}$ degeneracy, the corresponding shear strain along the $[\bar 12\bar 10]$ or $[10\bar 10]$ direction should be smaller than the estimated 2–3% shear strain.

The photoinduced strain generation can be caused by thermoelastic, electron-acoustic deformation potential, or the inverse piezoelectric effect (IPE). Both the thermoelastic and deformation potential mechanisms produce small stresses (<10^−3^ GPa, see Supplementary Note 7) due to the small excitation density; too small to induce the tens of meV band-gap shifts^47^ that we observe. We believe that the IPE is the most likely mechanism for shear strain generation. IPE has been observed in various piezoelectric semiconductors^49,50^ and is also expected in Te since it is an anisotropic crystal with moderately high piezoelectric coefficients^26,27,51^ and predicted to show ferroelectricity^52^. Upon pump excitation, photogenerated carriers screen the built-in electric field in the film (most likely at the surface)^27^ that induces a strain wave through IPE. The induced strain relaxes as excited carriers decay because the screening slowly diminishes. The photoinduced strain generation can be observed through a periodic change of the TR signal due to the interference between the propagating strain wave and the probing laser pulse^53,54^. We have observed a weak oscillatory component with an average period of 26.3 ± 0.1 ps in our TR signal corresponding to the coherent longitudinal acoustic phonons (CLAP) propagating in the film, which supports photoinduced strain generation in our samples (see Supplementary Note 7). The sound velocity obtained is much lower than the longitudinal sound velocity along the *c*-axis or perpendicular to it, and rather closer to the velocity of the shear wave along the same directions. This may be an indication of shear strain contributions along with other modes in our samples^55,56^.

### Polarization and energy-dependent carrier dynamics

**Fig. 5:**
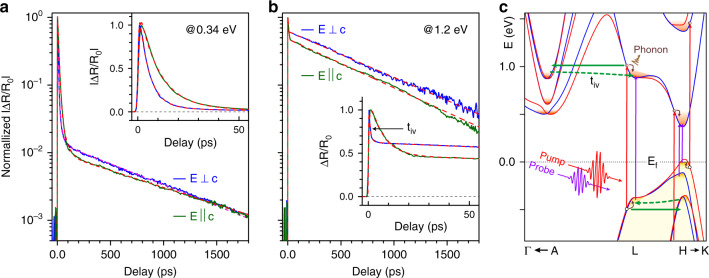
Polarization and energy-dependent carrier decay dynamics. Representative polarization-resolved TR (Δ*R*/*R*_0_) traces of Te samples around the fundamental gap **a** and higher energy transition region **b**. Dashed red lines are the multi-exponential fits of each corresponding data over a long delay range, as described in the main text. Insets show the initial decay responses of each corresponding trace. Around the band-edge region, a majority of the TR signal relaxes within first 30 ps due to ultrafast intraband thermalization followed by interband recombination. Around the higher energy transition, only a fraction of the maximum TR signal decays rapidly, predominantly by intervalley scattering accompanied by intraband thermalization, while the rest of the signal recovers on the order of a nanosecond due to interband recombination. **c** Schematic drawing of carrier excitation and relaxation processes within the extended bulk band structure of Te around high symmetry points in the Brillouin zone. Pump excitation is indicated by vertical red arrows and probe excitation is shown by vertical purple arrows. Intervalley forward and return scattering processes are also shown by green solid and dashed arrows.

The transient polarized TR spectra enable study of multiple carrier decay processes. Polarized time decays from the low and high-energy regimes are shown in Fig. 5a, b. Simple tri-exponential decay functions convoluted with a Gaussian response function (see Eq. 2 in Supplementary Note 3) fit the data very well over the entire delay range and provide a quantitative picture of overall decay behavior. Near the band-gap region (Fig. 5a), the initial fast and slow decay constants are $\tau _1^ \bot \sim 5 \pm 1\,{\mathrm{ps}}$ and $\tau _2^ \bot \sim 20 \pm 1\,{\mathrm{ps}}$, respectively, for $E \bot c$. The initial fast and slow decay times are twice as long for $E||c$, i.e., $\tau _1^{||}\sim 9\,{\mathrm{ps}}$ and $\tau _2^{||}\sim 36\,{\mathrm{ps}}$, respectively. There is also a small (~1%) residual signal that slowly decays within $\tau _3^ \bot \sim \tau _3^{||} \sim 1\,{\mathrm{ns}}$. Around the high-energy regime (Fig. 5b), only a fraction (~30%) of the peak intensity decays abruptly within a sub-ps ($\tau _1^{||}\sim 0.8 \pm 0.1\,{\mathrm{ps}}$) time for $E \bot c$, and is nearly 10-times slower for $E||c$. The majority of the signal at high-energy decays slowly with a decay time on the order of ~1 ns for both polarizations.

The physical origin of the TR decays can be understood from the schematic band diagram in Fig. 5c. A few femtoseconds after pump excitation (vertical red arrows), the TR response decreases abruptly due to absorption bleaching through state filling of hot carriers before a Fermi-Dirac distribution is established by carrier-carrier scattering. Subsequent recovery of the TR response occurs through basic carrier relaxation processes that vary at different bands due to the different scattering channels involved. The TR signal near the band-gap decays rapidly as hot carriers thermalize quickly with the lattice through a cascade of optical phonon emissions and carrier-carrier scattering, as indicated by curved arrows in Fig. 5c. The early transient is dominated by thermalization of hot holes due to efficient scattering with the cold degenerate hole gas due to their higher effective mass (*m*_h_ > *m*_e_). The fast decay of $\tau _1^ \bot \sim 5\,{\mathrm{ps}}$ corresponds to an upper bound to the effective scattering time for a hole density of *N*_d_. The subsequent slower decay $\tau _2^ \bot \sim 20\,{\mathrm{ps}}$ is attributed to the interband recombination near the band-gap. Since our samples are thinner than the laser penetration depth *l*_IR_ in Te around the IR-region ($l_{{\mathrm{IR}}}\sim 50\,{\mathrm{nm}}$)^28^, carrier diffusion processes do not contribute to the decay response. The weak residual signal (~1% of the peak) persists over nanosecond times and is attributed to feeding of long-lived carriers from higher lying bands through phonon-assisted intervalley processes. The initial rapid decay for $E||c$ is a factor of two slower than that for $E \bot c$, as shown more clearly in the insets of Fig. 5a. This observation is directly related to the anisotropic transport characteristics of Te. The electrical conductivity of bulk Te, including the hole mobility and scattering time, has been found to be larger (~1.2–2.3 times larger) along the *c*-axis than that of perpendicular to the *c*-axis^15,57^. In particular, the free carrier scattering time parallel to the *c*-axis is measured to be three-times longer than that perpendicular to the *c*-axis due to stronger coupling of polar optical scattering perpendicular to the *c*-axis, which is the main scattering process in *p*-doped Te samples^15,57^. Such an anisotropic carrier scattering process is nicely reflected in the carrier dynamics of our samples, which could be also relevant in other anisotropic materials.

The carrier decay dynamics at high energies is associated with the direct transition near the band-edge at the L-point, and therefore involves different scattering processes. Due to closely spaced CBs at the A-valley (Fig. 5c), intervalley coupling of hot carriers between the L- and A-valley is highly favorable. Photoexcited hot carriers at the L-valley will scatter rapidly to the A-valley via deformation potential scattering followed by return scattering to the L-valley, as indicated by green solid and dotted arrows, respectively, in Fig. 5c. The TR response for $E \bot c$ displays a sharp transient within a sub-ps time ($\tau _1^ \bot \sim 0.8\,{\mathrm{ps}}$) followed by slower recovery of the signal ($\tau _2^ \bot \sim 5\,{\mathrm{ps}}$) until it reaches thermal equilibrium with the lattice (quasi-steady state regime). We attribute the early sub-ps decay time to an upper limit of average intervalley scattering time *τ*_iv_ between the L- and A-valleys and subsequent slower decay constant to the intraband cooling time within the L-valley, which is nearly identical with the band-edge thermalization time at the H-point. Our data of $\tau _{iv}\sim 0.8$ ps is comparable with the data obtained from other semiconductors such as GaAs^58^. Since the intervalley return scattering is much longer than the forward scattering due to the lower density of states at the A-valley, the second decay component $\tau _2^ \bot$ may involve both intervalley return scattering as well as intraband cooling processes. The TR signal after cooling of carriers, i.e., the degree of state filling of thermalized carriers at the band-edge, reduces only marginally (only ~30% of the peak), which is likely due to the reduced density of optically coupled state filling at the band minima due to dominant intervalley forward scattering. The ultrafast intervalley process creates a long-lived reservoir of carriers at the A-valley, which eventually bleeds back to the band minima at the H-valley. This extraction time corresponds to the long-lived decay ($\tau _3^ \bot \sim 1\,{\mathrm{ns}}$) seen for the H-valley transition (see Supplementary Note 4) The TR response for $E||c$ qualitatively resembles the subsequent long-lived decay response (Fig. 5b). The early decay is dominated by the much slower ($\tau _1^{||}\sim 8\,{\mathrm{ps}}$) carrier thermalization rate, which is again, as discussed earlier, supported by the arguments of higher mobility and scattering lifetime of free carriers along the *c*-axis. This observation of longer early decay response suggests intervalley scattering is effectively suppressed with $E||c$ and hints also at possible consequences of photoinduced band modification even in the higher lying bands.

## Discussion

Optical transitions in Te are found to be highly anisotropic and strongly perturbed upon photoexcitation, which are linked to its unique spin texture and band dispersion. The detailed analysis of transient reflectivity spectra at late times reveals direct degenerate transitions at the band gap between the Fermi level, 30 meV below the top of the *H*_4_ band-edge, and the $H_6^{CB}$ for both polarizations and a direct transition between $H_5 \to H_6^{CB}$ only for $E||c$, which agree well with bulk absorption measurements^15^. While such a result has been seen by others^15,31^, it is inconsistent with the group theory result that the $E||c$ transition should be forbidden at the fundamental gap^28^. This disagreement may reflect the unique spin texture and complex band dispersion near the band-edge. The data also shows an indirect transition between the doubly degenerate $H_6^{VB} \to H_6^{CB}$ at ~0.7 eV, which has been predicted theoretically but not previously observed experimentally. In addition, we also identify a possibly direct transition at ~1.2 eV at the L-point band-edge. Detailed energy-dependent time traces show ~99% of the photoexcited carrier decay through recombination at the band-gap within 30 ps while ~1% of the photoexcited carriers are scattered from the VB or CB at the L-point to a remote valley likely at the A-point in the Brillouin zone. These trapped carriers gradually feed from the remote valley over the following 1 ns back to the CB and VB band-edges at the H-point where they rapidly recombine. These highest energy transitions appear to be long-lived, suggesting they can be a source for charged carriers, potentially important in the design of efficient thermoelectric and opto-electronic devices.

The detailed analysis of TR spectral dynamics reveals ~2% photoinduced shear strain through IPE, which breaks the screw symmetry of the Te and lifts the degeneracy of the $H_6^{CB}$ (~20 meV splitting). This causes also a ~30 meV decrease in the spin-split gap between the *H*_4_ and *H*_5_ VBs due to strain-induced reduction in the SOI. The bands relax to equilibrium as the photoexcited carriers relax within 30 ps. These results are confirmed by detailed ab initio DFT calculations as a function of strain, which indicate that the photoinduced shear strain is ~2%. While such a strain is too low to achieve a topological phase transition^8,24^, these results provide solid evidence that it is possible to manipulate symmetry protected energy states through photoexcitation. We find that even relatively modest photoinduced shear strains in Te can lift the degenerate crossing of the Weyl point at the $H_6^{CB}$. Future research into the response of quantum materials (e.g., Weyl semimetals) to intense illumination may be quite promising.

## Methods

### Growth of Te nanosheets

Te nanosheet samples were grown chemically through the reduction of sodium tellurite (Na_2_TeO_3_) by hydrazine hydrate (N_2_H_4_) in an alkaline solution at temperatures 160–200 °C with the presence of crystal-face-blocking ligand polyvinylpyrrolidone (PVD)^12^. The hydrophilic Te nanosheets were transferred to thermally oxidized Si substrates by the Langmuir–Blodgett assembly process.

### Sample characterization

The surface morphology and thickness of Te sheets were measured by using atomic force microscope (AFM). Measurements were conducted in air on a Veeco AFM using gold (Au) coated platinum (Pt) cantilevers operating in tapping mode. Raman spectroscopy was applied to characterize sample quality and crystal orientation. The measurements were performed using a Raman microscope with 632.8 nm laser excitation in the backscattering configuration under ambient condition. The Te sample was rotated with respect to the laser polarizations in order to identify the crystal *c*-axis and characterize polarization dependent Raman spectra. A ×100 objective was used to focus the incident beam and collect the scattered signal and then dispersed by 1800 g/mm grating after the resonant scattered laser light is removed using a double subtractive mode spectrometer. The Raman signal is measured with a spectral resolution of 1 cm^−1^. Laser power was maintained ~100 μW during the measurements to minimize laser-heating effect in our samples.

### Transient reflectance spectroscopy

Ultrafast pump-probe TR measurements were performed based on a Ti:sapphire oscillator which produces 150 fs pump pulses at a central wavelength of 800 nm with 80 MHz repetition rate and an average power of 4 W. The majority (80%) of the laser beam was used to pump an optical parametric oscillator (OPO), which generates signal (0.8–1.2 eV) and idler (0.3–0.72 eV) photons, which can be tuned continuously over a wide range. All measurements were performed using 820 nm ($\hbar \omega _p = 1.51\,{\mathrm{eV}}$) as the pump pulse and both signal and idler outputs as probe pulses, respectively, as illustrated in the Supplementary Fig. 3. The polarization of the probe pulses with respect to the crystal *c*-axis is controlled using a CaF_2_ double Fresnel rhomb on the probe beam path. The probe pulses are delayed in time with respect to the pump pulses by using a motorized linear translation stage. The pump and probe beams are spatially overlapped on a single Te sheet using a ×40 protected silver reflective objective with the help of a CCD camera, and the reflected beams are directed to the liquid nitrogen cooled InSb detector. The pump beam is filtered out during the TR measurements using a long pass filter. The pump-induced signal is collected with a lock-in amplifier phase-locked to an optical chopper that modulates the pump beam at a frequency of 1 kHz. The focused spot size of both the pump and probe beams is nearly identical, i.e., ~2 μm in diameter. An average pump fluence of ~0.5–1 mJ/cm^2^ was used throughout the experiments, while the probe fluence was kept nearly one order of magnitude lower.

### Strain-induced band structure calculations

The calculation of the Te band structure under strain is performed based on density functional theory (DFT) as implemented in the Vienna ab initio Simulation Package (VASP)^59^. The projector-augmented wave method^60,61^ is employed for DFT calculations, with the Perdew–Burke–Ernzerhof (PBE)^62^ generalized gradient approximation (GGA)^63^ for the exchange–correlation functional. The spin-orbit coupling is considered in all DFT calculations. The Tkatchenko and Scheffler (DFT-TS) method^64^ was employed for the van der Waals correction which provides excellent agreement with experimental lattice constants of the bulk Te^52,65^. Other van der Waals correction methods such as DFT-D2^66^ and DFT-D3^67^ were also used to check against the DFT-TS results but negligible differences are found, and therefore, not reported in this paper. The Г-centered Monkhorst-pack^68^ k-point sampling is used for the Brillouin zone integration. We find that the 10 × 10 × 8 grid is sufficient for convergence. The plane-wave cutoff energy is set to 500 eV and the convergence criterion for electronic relaxations is set to 10^−6^ eV. All atoms in the computational cell are relaxed until the Hellmann–Feynman forces are <0.01 eV Å^−1^. The band structures were obtained using the HSE06 functional^69,70^.

## Supplementary information

Supplementary Information

## Data Availability

The data that support the plots within this paper and other findings of this study are available from the corresponding authors upon reasonable request.
