# Supplementary material for: Ultrafast photoinduced band splitting and carrier dynamics in chiral tellurium nanosheets
Source: Nat Commun. 2020 Aug 10;11:3991. doi: 10.1038/s41467-020-17766-5 (PMC7417742; doi:10.1038/s41467-020-17766-5)
Supplement: Supplementary file 1 — Supplementary Information [file 41467_2020_17766_MOESM1_ESM.pdf]

Supplementary Information

Ultrafast Photoinduced Band Splitting and Carrier Dynamics in Chiral Tellurium Nanosheets

Giriraj Jnawali *[1], Yuan Xiang [2], Samuel M. Linser [1], Iraj Abbasian Shojaei[1], Ruoxing Wang [3], Gang Qiu [4], Chao Lian[5], Bryan M. Wong[5], Wu Wenzhuo [3], Peide D. Ye [4], Yongsheng Leng [2], Howard E. Jackson[1] and Leigh M. Smith*[1]

[1] Department of Physics and Astronomy, University of Cincinnati, Cincinnati, OH 45221, USA
[2]Department of Mechanical & Aerospace Engineering, The George Washington University, Washington, D.C. 20052, USA
[3] School of Industrial Engineering, Purdue University, West Lafayette, IN 47907, USA
[4]School of Electrical and Computer Engineering, Purdue University, West Lafayette, IN 47907, USA
[5] Department of Chemical & Environmental Engineering, Materials Science & Engineering Program, University of California, Riverside, Riverside, CA 92521, USA

**Corresponding authors*

Supplementary Note 1: Strain effects on the electronic band structure of Te

A. Band structure under zero strain

The Te band structure is calculated using density functional theory (DFT) as implemented in the Vienna ab initio Simulation Package (VASP).[1] Computational details are described in Methods section of the main text. Supplementary Figure 1 shows the calculated Te band structure around high symmetry points in the Brillouin zone. All the features in the band structure are reproduced by our calculations (see main text for details). In particular, the computed band gap is at the H-point with the magnitude of 0.25 eV, which is somewhat lower than the experimental value of 0.32 eV but is in good agreement with other DFT results.[2, 3] The two-fold spin degeneracy of the H_6 conduction band (CB) or conventionally H_6^{CB} due to the three-fold screw symmetry of Te is clearly seen. There are two non-degenerate (spin-polarized) H_4 and H_5 valence bands (VBs) with splitting gap of about 0.14 eV, which is consistent with our observation of the energy-splitting between the H_4 and H_5 band edges, i.e., $\Delta E = 0.115$ eV, as well as recent angle-resolved photoemission spectroscopy (ARPES) mapping of VBs of single crystal Te.[4] Not only the low-energy bands but also higher lying bands such as H_6 VB or H_6^{VB} as well as L- and A-valley structures are also consistent with previous DFT studies, which are explicitly discussed in the main text. Next, we discuss about how these features, in particular near the Fermi energy around the H-point, are affected by the strain applied in the Te crystal along different crystallographic directions.

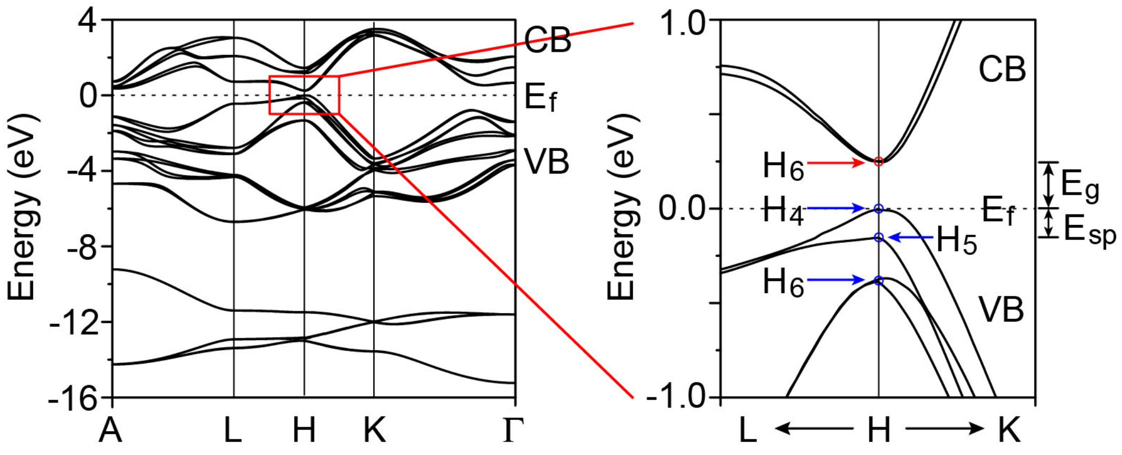

Supplementary Figure 1 | Computed band structure of Te under zero strain. The band structure is calculated along the symmetry directions of the Brillouin zone of the bulk Te. The Band structure is particularly relevant around the H-point near the Fermi level E_f where the band-gap E_g exists, as shown by the enlarged diagram on the right. The entire band features, including the two-fold spin degeneracy at H_6 CB, the spin-polarized non-degenerate frontier H_4 and H_5 VBs (with spin-split gap E_{sp}), the two-fold spin degeneracy of H_6 VB, as well as the slightly indirect band-gap between H_6 CB and H_6 VB along the H-K direction, are consistent with previous works.[2, 3, 5, 6]

B. Band structure under uniaxial strains or a hydrostatic pressure applied

It is well known that the band structure of a solid crystal is modified by inducing strains. The band modification, however, depends on how the crystal symmetry is affected by different types of strains. In the case of Te, an anisotropic chiral semiconductor, its complex band structure near the band-edge displays unique strain effects that have been demonstrated by recent studies of ab initio electronic structure calculations.[2, 3, 6] Applying uniaxial strains or a hydrostatic pressure on a Te crystal (see Supplementary Figure 2a), we see that the band-gap near the H-point in the Brillouin zone increases in the case of a compressive strain applied and decreases in the case of a tensile strain or a hydrostatic pressure applied. Very large tensile strains or hydrostatic pressures can lead to the closing of the band-gap or even a band crossing of the frontier bands (band inversion). However, the three-fold symmetry of the helical structure is preserved and thus the two-fold degeneracy of the H_6^{CB} band is maintained. These results are shown in Supplementary Figure 2b.

When a shear strain is applied, however, the screw symmetry of the helical chains is broken, which lifts the degeneracy of H_6^{CB}, as well as reduces the spin-split gap of the uppermost VBs in the vicinity of H-point. We have discussed this scenario in the main text.

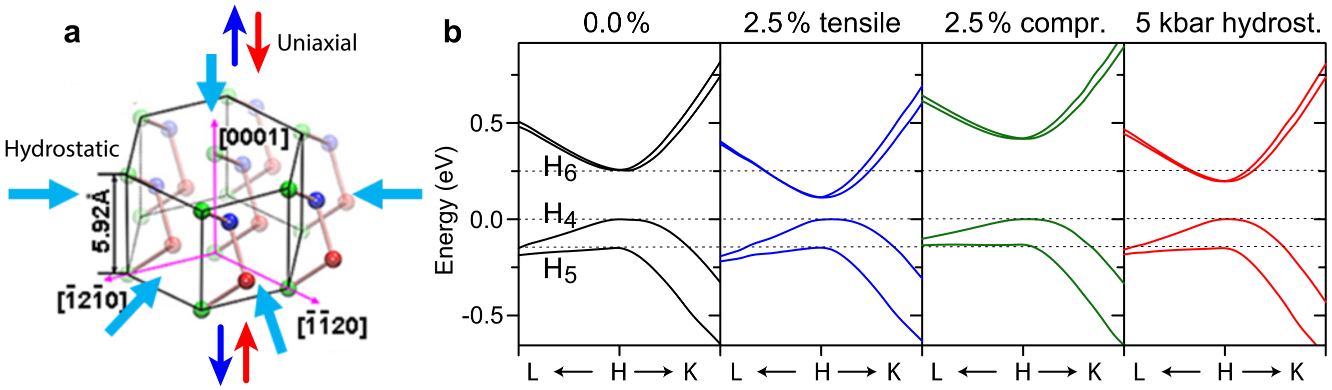

Supplementary Figure 2 | Computed band structures of Te under uniaxial strains or a hydrostatic pressure. **a** Schematic of various strains applied along different directions of the Te crystal. The dark blue and red arrows show the tensile and compressive uniaxial strains applied along the c-axis, respectively. The light blue arrows show a hydrostatic pressure applied on the crystal from all directions. **b** Changes of the band structure around the H-point near the Fermi level without and with various strains (plotted in different colors). The types and magnitudes of the strains are also shown on the top of each plot.

Supplementary Note 2: Polarized transient reflectance spectroscopy setup

The standard pump-probe setup used for polarized transient reflectance spectroscopy measurements is shown schematically in Supplementary Figure 3. The Si/SiO_2 substrate with the Te nanosheet is mounted to the 10 K cold finger of a microscope-cryostat. A short 1.51 eV pump pulse (150 fs) is superimposed collinearly with a probe pulse (150 fs) of variable photon energies $(0.3 - 1.2\ \text{eV})$ and focused onto a single Te nanosheet using a $40 \times 0.5\ \text{NA}$ reflective objective. The electric field polarization (ϕ) of the incident probe beam is controlled using a CaF_2 double Fresnel Rhomb rotator on the probe beam path. Measurements are performed with probe polarization either parallel ($E \parallel c$), i.e., $\phi = 0°$ or perpendicular ($E \perp c$), i.e., $\phi = 90°$ with respect to the c-axis of the Te crystal. The crystal c-axis is defined based on polarization dependent Raman measurements on a single Te nanosheet samples. For steady-state reflectance measurements, the probe reflectance is detected with pump off conditions. For pump-induced transient reflectance measurements the probe reflectance is detected with pump on conditions in which the probe pulses are delayed in time with respect to the pump pulses by using a motorized linear translation stage. The reflectance signal is detected using the liquid nitrogen cooled InSb detector. The probe pulses are delayed in time with respect to the pump pulses by using a motorized linear translation stage. The pump beam is filtered out during the TR measurements using a long pass filter. The pump-induced signal is collected with a lock-in amplifier phase-locked to an optical chopper that modulates the pump beam at a frequency of 1 kHz.

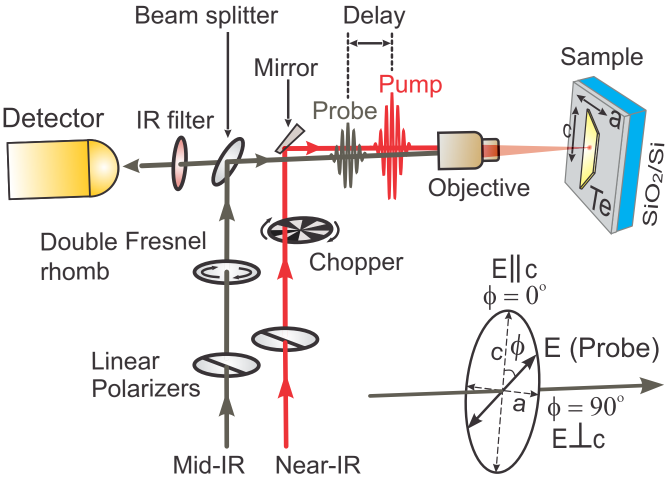

Supplementary Figure 3 | Polarized transient reflectance spectroscopy (TRS) setup. Simple illustration of polarized TRS experiment performed in Te nanosheets, which are deposited on SiO_2/Si substrate. The polarization state of the probe beam with respect to the crystal c-axis is controlled by using a double Fresnel rhomb. Time and probe energy dependent pump-induced reflectance spectra are measured at each orthogonal polarizations, as shown on the bottom right, using standard pump-probe detection technique.

Supplementary Note 3: Transient reflectance spectroscopy (TRS) at 300 K

A. Optical transitions at 300 K

Supplementary Figure 4a displays a false color map of the 300 K TR spectra measured over a wide spectral region ($0.3 - 1.2$ eV) for the probe polarized parallel ($E \parallel c$) and perpendicular ($E \perp c$) to the c-axis. Overall, the TRS map exhibits strong anti-symmetric features around low-energy ($0.3 - 0.5$ eV) and high-energy ($0.8 - 1.2$ eV) regions and a relatively weaker and broader signal near the mid-energy ($0.5 - 0.8$ eV) region, corresponding to a series of interband optical transitions. The map also shows that the strong features remain essentially unchanged in shape with time except for their magnitudes, suggesting only a weak perturbation of the transition response by the carrier thermalization process. These features are more apparent in Supplementary Figure 4b where the spectral slices at 50 ps delay from each polarization are plotted (blue circles). The derivative-like spectral features of the transient signal around the low- and high-energy regions exhibit significant differences for the two polarizations, while the broad feature in the mid-energies also displays some sensitivity to polarizations. With $E \parallel c$, a nearly overlapped additional feature is observed at $\sim$0.44 eV, which is not present with $E \perp c$, suggesting the polarization sensitive optical absorption in the Te samples.

It is known that the TR response is related to the perturbation of the dielectric function of the material induced by photoexcitation of charged carriers. For the Lorentzian form of the dielectric function under low-field modulation with the parabolic band approximation, TR spectra around the band-edge regions can be analyzed by using a derivative Lorentzian lineshape functional form appropriate for excitonic transitions,[7]

$$\frac{\Delta R}{R_0}(E) \backsimeq \sum_{j=1}^{n} Re\left[A_j e^{i\varphi_j}\left(E - E_j + i\Gamma_j\right)^{-2}\right], \quad (1)$$

where n represents the number of spectral functions for the possible interband transitions involved, E is the probe photon energy, and A_j, φ_j, E_j, and Γ_j are the amplitude, phase, transition energy, and the energy broadening parameter of the jth feature, respectively. In order to quantify each of these features and corresponding transition energies, the TRS spectra are fitted using Eq. 1, as shown with red dashed-lines in Supplementary Figure 4b. The spectra with $E \perp c$ can be fitted by three resonances ($n = 3$) while the spectra with $E \parallel c$ can be fitted by four resonances ($n = 4$). Moduli of the individual resonances, which have been obtained from Eq. 1, are also overlapped with minor vertical shifts for clarity (black solid lines in Supplementary Figure 4b). The values of the transition energies obtained from least square fitting are indicated by vertical dashed grey lines.

Now, we compare the transition energies of each jth feature with the detailed band structure around the H−point of the bulk Brillouin zone of Te,[5, 6, 8] as shown schematically in the main text. The lowest energy transition $H_4 \rightarrow H_6^{CB}$ with $E \perp c$ is $E_1^{\perp} = 0.34 \pm 0.002$ eV, which is nearly the same value as the low temperature value shown in the main text. This behavior nicely corresponds with the peculiar temperature dependent absorption coefficient in Te previously observed.[9] One can also see the lowest energy transition with $E \parallel c$, which is blue-shifted by ~ 20 meV to $E_1^{\parallel} = 0.36 \pm 0.002$ eV. Such a polarization anisotropy of optical transition at the band-gap remains unchanged over entire delays, in contrast to the strong modulation up to 30 ps observed at 10 K (see main text). The $E_1^{\perp}$ transition energy as well as the polarization anisotropy, i.e., the dichroism of the optical absorption edge, agree very well with previous results obtained by linear absorption measurements on degenerately p-doped Te samples at room temperature.[9-11] The optical transition between $H_5 \rightarrow H_6^{CB}$ is observable only with $E \parallel c$ and totally absent with $E \perp c$, which is in agreement with low temperature measurements and consistent with the expected dipole allowed transition between these two states. The $H_5 \rightarrow H_6^{CB}$ transition energy is estimated to be $E_2^{\parallel} = 0.44 \pm 0.005$ eV, which is again nearly identical with the value measured at 10 K. The spin-split VB gap or the separation between the H_4 and the H_5 VBs turns out to be $E_2^{\parallel} - E_1^{\perp} = 100$ meV. This value, taking into account the doping-induced Fermi level shift, is nearly the same as the so-called 11-micron hole absorption band previously measured in a bulk single crystal of Te.[12, 13] The energy difference between the $E_2^{\parallel}$ transition and $E_1^{\parallel}$ transition decreases to $E_2^{\parallel} - E_1^{\parallel} = 80$ meV, which suggests not only the fundamental gap but also the gap between spin-split VBs is anisotropic at room temperature. In addition to the direct transitions between uppermost VBs and the lowest CB, there is an indication of a higher energy transitions at around $E_3^{\parallel,\perp} = 0.75 \pm 0.005$ eV with the lineshape sensitive to polarizations. Since the TR signal is nearly one order of magnitude weaker than the near band-edge transition, this transition might be indirect in **k**-space, as expected from the band structure.

We also observed a higher lying transition at $E_4^{\perp} \sim 1.16 \pm 0.005$ eV, which is blue shifted by 20 meV to $E_4^{\parallel} \sim 1.18 \pm 0.005$ eV. Due to limitations of the spectral range of the probe pulse, a full derivative-like lineshape is not observed, which causes uncertainty in the precise energy transition. Nevertheless, the transition is clearly sensitive to polarizations and the anisotropy is the same as in the fundamental gap. This high-energy transition is noticeably red-shifted (by ~ 80 meV for $E \perp c$ and ~ 60 meV for $E \parallel c$) as compared to the value observed at low temperature $E_4^{\parallel,\perp} \sim 1.24 \pm 0.005$ eV, suggesting fundamentally different in nature. Since the CBs above H_6^{CB} and VBs below H_6^{VB} at the H-point are far apart, the next higher energy transition at the H-point beyond $E_3^{\parallel,\perp}$ is not accessible with our

probe beam. Therefore, we attribute the transitions $E_4^{\perp}$ and $E_4^{\parallel}$ are caused by a direct transition at the L-point. Detail understanding of higher energy transitions in Te requires further theoretical calculations.

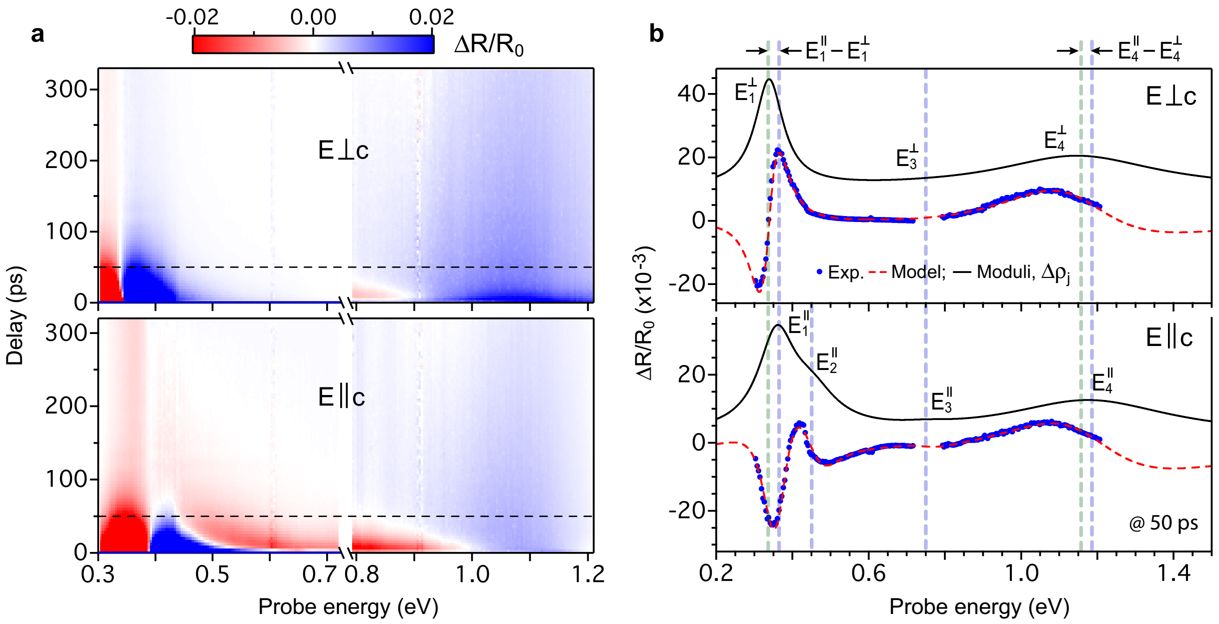

Supplementary Figure 4 | Polarized transient reflectance response of Te nanosheets around the band-edge region. a Two-dimensional false color map of pump-probe delay dependent transient reflectance spectra $[\Delta R/R_0\,(E,t)]$ over an extended probe energies for parallel $E \parallel c$ and perpendicular $E \perp c$ relative to the c-axis of Te crystal. Narrow region of probe energies between $0.72 - 0.8$ eV is missing due to technical limitations. Negative signal (red) indicates a pump-induced increase in absorption and positive signal (blue) corresponds to decrease in absorption. Overall, strong features with distinctive dynamics at different energies are clearly visible, suggesting a series of optical transition with some polarization anisotropy. **b** Transient spectra (blue spheres) acquired at 50 ps time delay with two orthogonal probe polarizations. Each spectrum is fitted with a simple model described in the main text (red dashed lines). Calculated moduli $\Delta\rho_j$ of each fit are also plotted with vertical offset (black lines). Both the model fits and the moduli show transient features corresponding to the series of direct and indirect interband transitions in Te, as indicated by vertical dashed lines. The transition energy for $E \parallel c$ is blue-shifted by $\sim$20 meV, suggesting band-edge optical anisotropy in Te at room temperature.

B. Carrier dynamics at 300 K

Polarization dependent carrier decay processes are investigated by measuring TR time traces at different probe energies following excitation of the sample with 1.51 eV pump pulses. Representative polarized time traces taken from the nanosheet at 300 K from two low- and high-energy regimes are shown in Supplementary Figure 5a,b. At energies intermediate between these two regimes, the TR response is very weak and fast due to lack of any direct optical transitions and, therefore we will not discuss it here. Near the band gap region (Supplementary Figure 5a), the majority of the TR decays exponentially within first 30 ps followed by a very weak (nearly 2-orders of magnitude lower than the

peak) residual signal, which persists over 300 ps. Around the high-energy regime (Supplementary Figure 5b), the time traces display initial ultrafast decay of the signal followed by a long exponential recovery of the remaining signal at later times. In order to quantify the overall decay behavior around the low and high energy regimes, each time trace is fitted using multi-exponential functions convoluted with a Gaussian response function:[14]

$$\Delta R/R_0\,(t) = \sum_{i=1,2,3} c_i \cdot \sigma \cdot exp[(\sigma/2\tau_i)^2 - (t/\tau_i)] \cdot [1 - erf\{(\sigma/2\tau_i) - (t/\sigma)\}], \tag{2}$$

where c_i is the amplitude with decay time constant of τ_i of the ith exponential term and σ is full width half maximum of the pump laser pulse ($\sigma = 200\ fs$). A least square fitting to Eq. (2), as shown by dashed red lines in Supplementary Figure 5a,b estimates the decay time constants τ_i of each ith decay channel at different bands. Near the low-energy region, the decay constant of the majority of the signal is $\tau_1^{\perp} \sim 17 \pm 1$ ps for $E \perp c$ and $\tau_1^{\parallel} \sim 22 \pm 1$ ps for $E \parallel c$, respectively, followed by long-lived ($\tau_2^{\perp}$, $\tau_2^{\parallel} \gtrsim 500$ ps) residual signal. Around the high-energy region, only a fraction of the peak intensity decays abruptly within a few ps ($\tau_1^{\perp} \sim 10 \pm 1$ ps for $E \perp c$ and $\tau_1^{\perp} \sim 12 \pm 1$ ps for $E \parallel c$) and the remaining signal decays rather slowly ($\tau_2^{\perp}, \tau_2^{\parallel} \gtrsim 300$ ps) for both polarizations. The fractional signal of rapid decay is $\sim 50\ \%$ of the peak signal for $E \perp c$ while it is only $\sim 10\ \%$ of the peak signal for $E \parallel c$. Such differences of decay dynamics with respect to the polarization and energy of the probe laser beam are qualitatively similar as observed at low temperature (see main text).

Around the low-energy region, ultrafast carrier thermalization by carrier-carrier and carrier-phonon scattering is not distinguishable at room temperature. Therefore, the decay time constants of $\tau_1^{\perp}$ and $\tau_1^{\parallel}$ for both polarizations are attributed to the interband carrier recombination time including carrier thermalization. Due to ultrathin samples, thinner than the penetration depth l_{IR} in Te around the IR-region ($l_{IR} \sim 50$ nm),[9] carrier diffusion does not play a role in the decay dynamics. The subsequent weak residual signal (less than 2 % of the peak) observed after the recombination is attributed to a constant feeding of carriers from higher lying bands through phonon-assisted intervalley processes. Around the high-energy region, the polarized TR responses display sharp transient within ~ 10 ps, which is attributed to intervalley scattering followed by the intraband cooling of carriers via carrier-carrier and carrier-phonon scattering. Since the signal after initial rapid decay is reduced substantially with $E \perp c$ as compared to the signal with $E \parallel c$, this observation suggests intervalley scattering is effectively suppressed with $E \parallel c$. Subsequent slower decay of the signal is caused by persistent feeding of intervalley scattered carriers at neighboring valleys as well as carriers at higher lying bands to the H-

valley. Note that the initial rapid recombination time at all spectral regions is noticeably slower with $E \parallel c$ as compared to the times with $E \perp c$. This behavior is more apparent at low temperature (see main text), which is consistent with anisotropic carrier scattering times and hole mobility observed in Te crystal.[10] Overall, carrier decay dynamics at room temperature is qualitatively similar to the low-temperature dynamics, which has been described with sufficient detail in the main text.

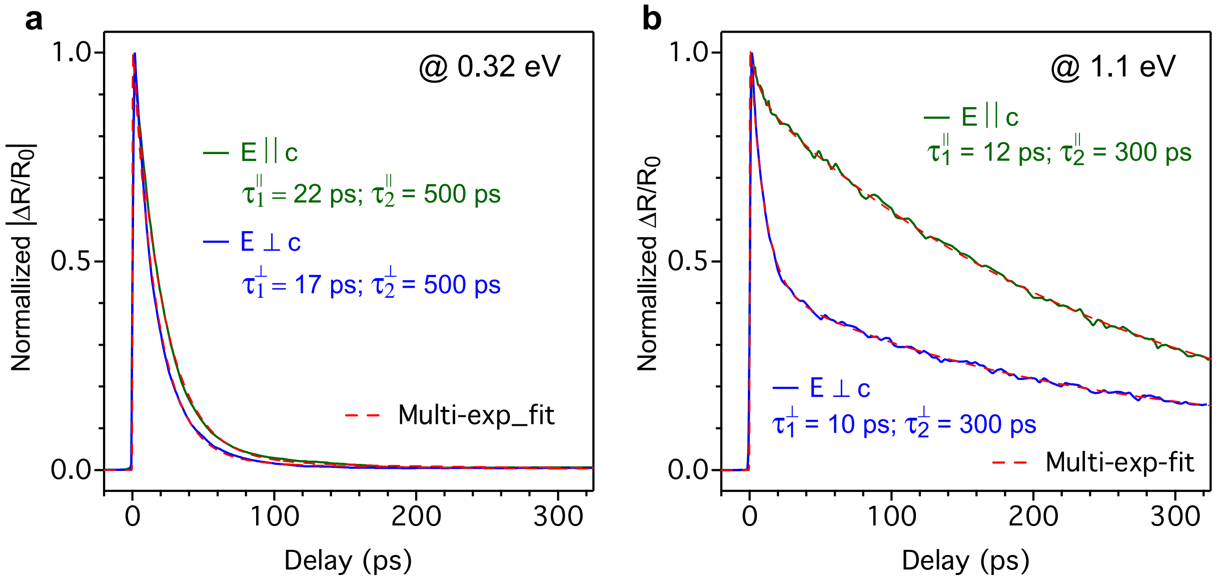

Supplementary Figure 5 | Polarized transient reflectance response of Te nanosheets at room temperature. Representative polarization resolved transient reflectance (TR) $\Delta R/R_0$ traces of Te samples around the fundamental band-edge **a** and high-energy transition region **b**. Dashed red lines are the multi-exponential fits of each corresponding data over a long delay range. Around the band-edge region, majority of the TR signal relaxes within first 30 ps due to ultrafast intraband thermalization followed by interband recombination. Around the higher energy transition, about half of the peak TR signal decays rapidly ($\tau_1^{\perp} \sim 10$ ps) with $E \perp c$ due to intervalley scattering accompanied by intraband thermalization. In contrast, TR signal with $E \parallel c$ decays only marginally (10 % of the peak) with a decay constant of $\tau_1^{\parallel} \sim 12$ ps, suggesting suppressed intervalley scattering and weak coupling to phonon. Rest of the signal for both polarizations decay rather slowly ($\tau_2^{\perp}, \tau_2^{\parallel} \gtrsim 300$ ps) due to constant feeding of carriers from higher lying bands to the H-valley.

Supplementary Note 4: Modeling carrier recombination dynamics at 10 K

One of the major results described in the paper is that a certain fraction of carriers at high energy scatter into remote valleys away from the H-valley minimum. These long-lived carriers then can provide a long-lived source for carriers which eventually recombine at the band edge. In order to make a more quantitative analysis of the decay dynamics in this system we perform modeling of time decays using coupled rate equations:

$$\frac{d}{dx}n_h(t) = G_0 - \frac{n_h(t)}{\tau_{h0}} - \frac{n_h(t)}{\tau_h};\ \frac{d}{dx}n_l(t) = G_1 - \frac{n_l(t)}{\tau_l} + \frac{n_h(t)}{\tau_h}, \tag{3}$$

where $n_h(t)$ and $n_l(t)$ denote the number density of thermalized carriers at the high (presumably the indirect L-valley) and low-energy (the lowest energy H-valley) regimes. Considering a two-level system, the first equation determines the scattering time τ_h of which describes the slow feeding of carriers from neighboring higher lying valleys to the H-valley as well as lifetime τ_{h0} of residual carriers in those higher lying indirect valleys,, which is beyond the experimental limit and assumed to be $\tau_{h0} = 5$ ns. In other words, we assume the lifetime of the indirect higher lying valleys is determined by feeding carriers to the H-valley band edge. The second equation describes the direct recombination time τ_l with accounting for constant feeding of carriers from higher energy bands to the band edge minima. The terms G_0 and G_1 determine the fraction of carriers excited into indirect higher lying valleys vs. the fraction excited into the H-valley minima. The solution of Eq. (3) fits the experimental time traces around both regions reasonably well, as shown by dashed red lines in Supplementary Figure 6a,b. We determine that the ratio $G_0/G_1 \sim 1.6$ % as expected from the time decays. The fits, however, does not cover the early transients (below 10 ps), where the carrier relaxation mostly dominated by carrier thermalization processes. The extracted values of carrier recombination times τ_l are , and ~15 ps and ~25 ps for $E \perp c$ and $E \parallel c$, respectively, and the lifetime of slow bleeding of carriers from higher lying valleys to H-valley for both polarizations is $\tau_h \approx 750$ ps. These values are nearly identical with the simple exponential fittings (shown in Supplementary Note 3) and agree with the previously described qualitative understanding of carrier decay dynamics in Te.

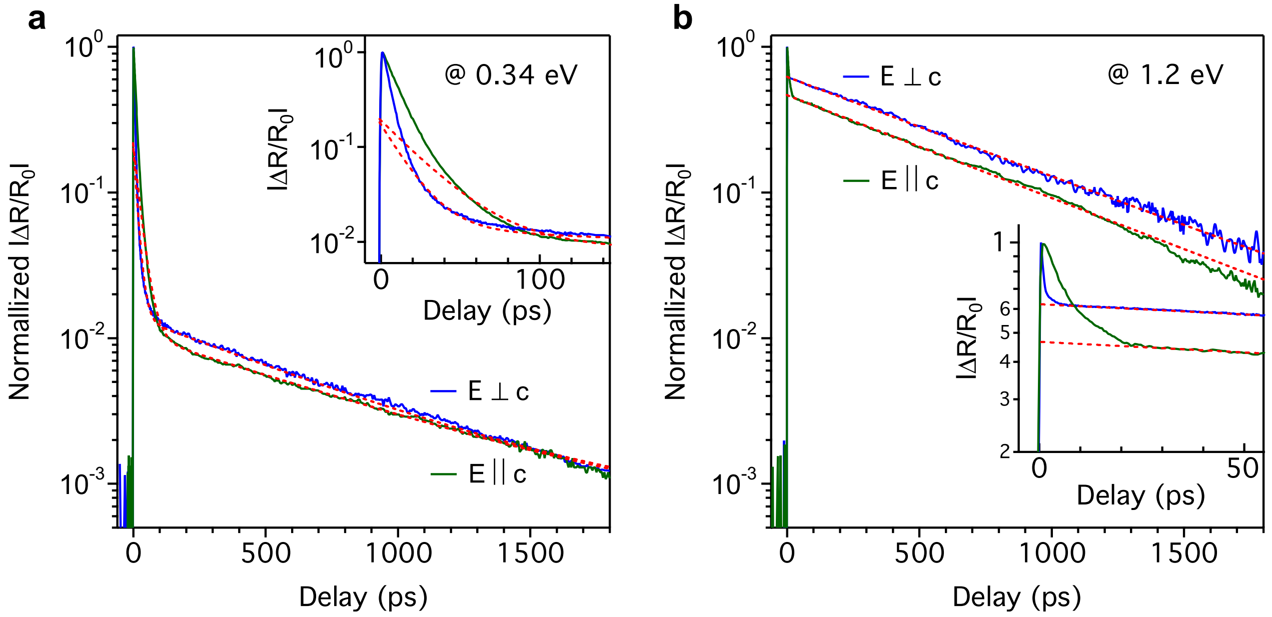

Supplementary Figure 6 | Polarization and energy dependent carrier dynamics. Representative polarization resolved transient reflectance (TR) $\Delta R/R_0$ traces of Te samples around the fundamental band-edge **a** and higher energy transition region **b**. Dashed red lines are the model fits of each corresponding data over a long delay range. Insets show zoom-in view, showing that the model does not cover early rapid carrier thermalization regime.

Supplementary Note 5: Modeling transient reflectance spectroscopy (TRS) data

For simplicity, our model of the transient reflectance response of Tellurium is based on the dominant contribution of band-filling due to interband photoexcitation of carriers. We constrain our calculations by considering the dynamics near the fundamental gap at H-point of bulk Brillouin zone of Te. The photoinduced band filling effect alters the dielectric response of Te, i.e., a fractional change in the complex refractive index ($\tilde{n} = n + ik$), and causes a change in absorption and reflectance of the samples.

Since the imaginary component of the complex refractive index, i.e., k, relates to the absorption coefficient α as: $k = \frac{\lambda}{4\pi} \cdot \alpha$, we first begin the model by calculating the photoinduced change in the absorption coefficient $\Delta\alpha$. The absorption coefficient α of semiconductors with parabolic bands can be described by the following expression:[15, 16]

$$\alpha(E, N_h, N_e, T) = \frac{C_\alpha}{E} \int_0^{E-Eg} \rho_c(E') \cdot \rho_\mathrm{v}(E' - E) \cdot \left[f_h\left(E - E_g - E', N_h, T\right) - f_e(E', N_e, T)\right] dE' \tag{4}$$

Where ρ_c and ρ_v denote density of states for the conduction and valence bands, N_h and N_e are the respective carrier densities for holes and electrons, f_h and f_e are the appropriate Fermi-Dirac distributions, T is the carrier temperature, and C_α is a constant factor fit to the data. Given the p-doped nature of our samples, we consider the total hole population N_h as a sum of doped holes N_d and photoexcited holes ΔN_h, i.e., $N_h = N_d + \Delta N_h$. The electron population N_e is taken to be entirely photoexcited, i.e., $N_e = \Delta N_e$, and the photoexcited electron and hole densities are taken to be equal, i.e., $\Delta N_e = \Delta N_h$, on the basis of charge neutrality. The carrier temperature T is assumed to be equal for electron and holes. Now we calculate the photoinduced absorption coefficient $\Delta\alpha$ using relevant parameters, which are known for our samples (see Supplementary Table 1).

Once the photoinduced modulation in the absorption is calculated, we can derive the modulation of the real part of index of refraction, Δn, using the Kramers-Kronig relation:

$$\Delta n(E, N_h, N_e, T) = \frac{\hbar c}{\pi} \int_0^\infty \frac{\Delta\alpha(E', N_h, N_e, T)}{(E')^2 - E^2} dE' \tag{5}$$

where $\Delta\alpha$ is calculated from Eq. (4). Note that although the upper bound of the integral is infinite, $\Delta\alpha$ rapidly approaches zero above the band-edge and thus our theoretical description of the absorption need not include the high-energy regime.

Once we derive both the real part of refractive index and the absorption coefficient, we can connect to these parameters to evaluate the reflectance of the sample. Assuming normal incidence of the probe beam on the sample, which is the case in our experiment, the reflectance of the sample without excitation is given by:

$$R = \frac{(n-1)^2 + k^2}{(n-1)^2 - k^2} \quad (6)$$

The fractional change of reflectance induced by pump excitation (normalized by initial reflectance R_0 before exciting the sample) can be expressed (in first-order expansion) as following:

$$\frac{\Delta R}{R_0} \cong \left[\left(\frac{8n_o k_o}{((n_o+1)^2 + k_o^2)^2}\right) \cdot \Delta k(E, N_h, N_e, T)\right] - \left[\left(\frac{4(1 + k_o^2 - n_o^2)}{((n_o+1)^2 + k_o^2)^2}\right) \cdot \Delta n(E, N_h, N_e, T)\right] \quad (7)$$

Where n_0 and k_0 are the background values of the real and imaginary components of the complex index of refraction, respectively. We take n_0, for light polarized perpendicular to the c-axis, to be constant at the average value of 4.95 for our probe tuning range, based on measurements of single-crystal Tellurium.[17] We calculate $k_0 = (\lambda/4\pi) \cdot \alpha$ from Eq. (4) using background estimates for carrier density. All basic material parameters are tabulated in Supplementary Table 1.

We fit theoretical lineshapes from Eq. 7 to our experimental transient reflectance spectra by parameterizing the density of doped holes, density of photoexcited carriers, carrier temperature, band-gap energy, and an overall scaling factor. We numerically optimize these parameters to minimize the sum squared error for each spectrum taken at distinct delay times, keeping the time-independent parameters consistent. The resulting fit parameters are tabulated in Supplementary Table 2.

Supplementary Table 1: Basic material parameters of Te used for modeling.

**Parameters**	**Values**
Electron effective mass*, m_e^*[4]	0.091 m_e
Hole effective mass*, m_h^*[4]	0.137 m_e
Refractive index, n_0[17]	4.95

**Geometric mean accounting for anisotropy.*

Supplementary Table 2: Band parameters of Te extracted from modeling TR spectra.

**Parameters**	**@ 10 ps delay**	**@ 30 ps delay**	**@ 60 ps delay**
Carrier temperature, T_e	84 K	67 K	47 K
Photoexcited carrier density, ΔN_{eh}	1.0×10^{18} cm^{-3}	5.2×10^{17} cm^{-3}	2.9×10^{17} cm^{-3}
Quasi-Fermi Energy (holes), E_{fh}	0.031 eV	0.027 eV	0.026 meV
Fundamental band gap, E_g	0.319 eV	0.319 eV	0.319 eV
Doping Density, N_d	1.8×10^{18} cm^{-3}	1.8×10^{18} cm^{-3}	1.8×10^{18} cm^{-3}

Supplementary Note 6: Polarization anisotropy of optical reflectance in Te

The ground-state optical transitions can be studied by measuring the linear optical absorption or reflectance of the samples. Such measurements are sometimes quite challenging in substrate (high absorbing substrates) supported ultrathin samples due to dominant contributions from the substrate. Linear dichroism, i.e., polarization anisotropy of optical absorption or reflectance, otherwise, can be very useful to reduce the substrate contributions and enhance the sensitivity of detecting optical response of the sample, particularly in anisotropic crystals. Here, we demonstrate that it is indeed possible to observe band-edge absorption features in a typical reflectance measurement of Te nanosheet samples supported on ~ 350 nm SiO_2/Si substrate. We observe the response of clearly distinguishable ground-state optical transitions, which are otherwise hardly visible from the reflectance spectra of the sample with substrate.

Supplementary Figure 7 displays probe photon energy dependent optical reflectance anisotropy (RA) or polarization anisotropy of Te nanosheets measured at 10 K. The RA is deduced by measuring the difference in reflectance for light normally incident and linearly polarized along two orthogonal directions, i.e., parallel $E \parallel c$ and perpendicular $E \perp c$ to the c-axis of the Te crystal. The normalized RA is expressed by: $RA = (R_{\parallel} - R_{\perp})/(R_{\parallel} + R_{\perp})$, where $R_{\parallel}$ and $R_{\perp}$ denote the reflectance along $E \parallel c$ and $E \perp c$, respectively. The individual reflectance spectra of each polarization are also shown on the top. The anisotropy is particularly pronounced around the band-edge regions, which are shown by thick grey lines and also indicated by particular transitions in the band structure. First minimum coincides with the $H_4 \rightarrow H_6^{CB}$ transition between the uppermost H_4 VB to the lowermost H_6 CBs, i.e., $E_1 = E_1^{\perp}$ and $E_1^{\parallel}$. The next maximum matches with the $H_5 \rightarrow H_6^{CB}$ transition between the spin-split

lower H_5 VB to the H_6 CBs, i.e., $E_2 = E_2^{\parallel}$. The positive peak of the RA is due to 180° phase shift of this transition, which is dipole forbidden for $E \perp c$. Weak maxima around the other two higher energy transitions $E_3 = E_3^{\perp,\parallel}$ and $E_4 = E_4^{\perp,\parallel}$ can also be recognized in the spectra. Overall, these RA features are consistent with the features observed in transient reflectance spectra and further supports our estimations of ground-state optical transitions in our Te samples.

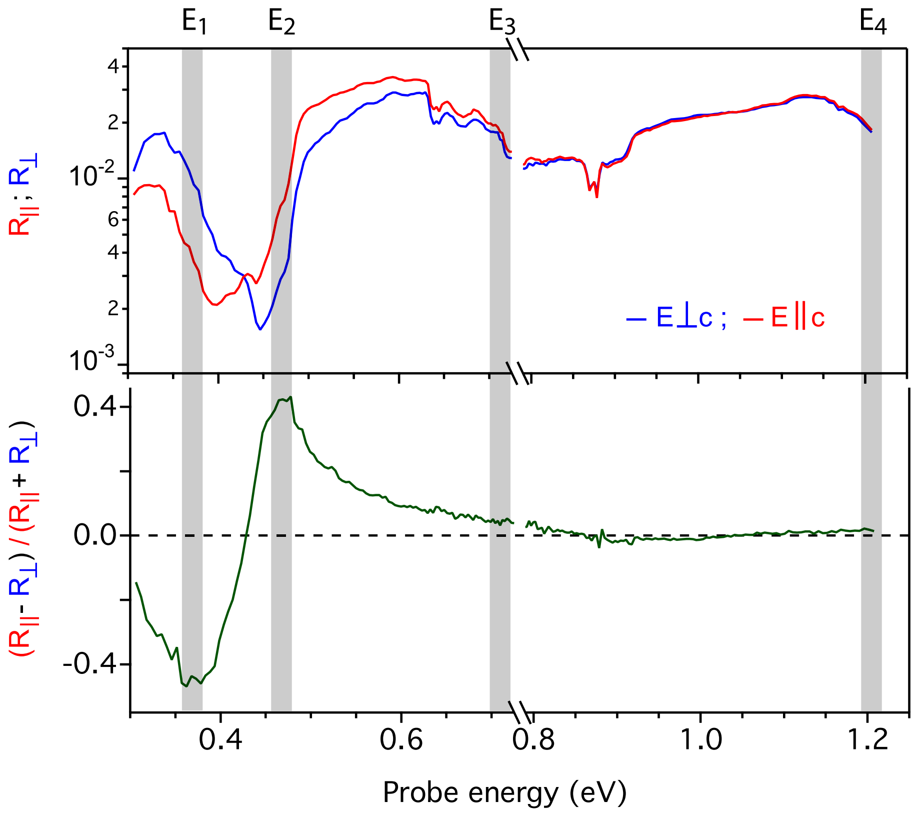

Supplementary Figure 7 | Polarization anisotropy near the band-edge of Te nanosheets. The polarization anisotropy (bottom plot) is calculated from the reflectance spectra (shown on the top) recorded for both $E \perp c$ and $E \parallel c$ polarizations. The anisotropy peaks near the band-edges of different bands around H-point of the Brillouin zone. Respective transition energies are also indicated with transparent grey thick lines. These transition energies are consistent with ground-state transitions extracted from the TR spectra (see main text).

Supplementary Note 7: Coherent longitudinal acoustic phonon in Te nanosheets

Coherent longitudinal acoustic phonons (CLAP) are propagating ultrasonic strain waves in solid crystals, which can be excited efficiently at the surface by ultrafast laser pulses through transient lattice deformation.[18-21] Since the strain wave propagation modifies the local dielectric properties of the materials, the induced oscillatory behavior of dielectric properties can be probed in the time-domain by various pump-probe spectroscopic techniques.[18, 22]

Supplementary Figure 8 displays probe wavelength dependent TR response measured following 1.51 eV pump excitation of the Te nanosheet samples and probing at different photon energies (wavelengths). Following initial ultrafast transients, the TRS signal oscillates in time and gradually decays because of interference between light waves reflected off the surface and light waves reflected off the propagating strain wave. The TR signal, therefore, contains both the electronic response of the material as well as the oscillatory interference signal due to the CLAP generated in the sample. In order to extract information about the oscillatory signal, each trace is fitted with following empirical function:

$$\frac{\Delta R}{R_0}(t) = A + \left(B \cdot e^{-\frac{t}{\tau_1}}\right) + \left(C \cdot e^{-\frac{t}{\tau_2}}\right) + \left\{D \cdot cos\left(\frac{2\pi t}{T} + \varphi\right) \cdot e^{-\frac{t}{\tau_{osc}}}\right\}, \quad (7)$$

where first three terms fit the non-oscillatory electronic response including bi-exponential (initial fast τ_1 followed by slower τ_2) response and last one fits the slowly decaying oscillatory signal. Coefficients B, C, D are amplitude of each decaying components and A is the overall background signal. Least square fitting method is applied to each wavelength dependent trace, as shown by black line superimposed on each trace, and useful parameters such as the velocity of the CLAP are extracted as fit parameters. An average amplitude of the oscillation is of the order of 10^{-4} and damping constant τ_{osc} varies from 50-100 ps. Interestingly, the oscillation period D remains unchanged for each probe wavelength. This behavior suggests that standing wave condition of CLAP propagation within the film is fulfilled, which is expected in ultrathin samples. The period of the oscillation is determined by film thickness d:

$$T = \frac{2 \cdot d}{\upsilon_S}, \quad (8)$$

where υ_S is the velocity of CLAP (sound velocity), which depends on the polarization of probing laser pulse. An average oscillation period of 26.3 ± 0.1 ps estimates the average velocity υ_0 of the LA phonon (sound velocity) of $\sim$ 1800 m/s in our ultrathin ($d \sim 24$ nm) Te samples. The estimated velocity υ_0 is lower than both the sound velocity along parallel (3400 m/s) and perpendicular to c-axis (2290 m/s), but higher than the velocity along xy-plane or shear plane (1390 m/s),[23] suggesting quasi-shear wave propagation in our sample.[24]

It is important to discuss the physical origin of the acoustic phonon generation in our Te samples. There are mainly three possible mechanism of stress generation upon photoexcitation: (1) thermoelastic stress (σ_{TH}), (2) electron-acoustic deformation potential stress, σ_{DP} and (3) inverse piezoelectric stress, σ_{IP}. The photoinduced σ_{TH} due to rapid lattice heating by electron-phonon coupling can be estimated by following the standard model,[19] $\sigma_{TH} = -3 \cdot \beta \cdot B \cdot \Delta T$, where β is the linear thermal expansion coefficient, B is the bulk modulus and ΔT is the maximum temperature increased by pump pulse. Due to

anisotropic crystal, Te has negative expansion coefficient parallel to c-axis, i.e., $\beta = -5\times10^{-6}\ K^{-1}$, and positive coefficient perpendicular to c-axis, i.e., $\beta = 5\times10^{-6}\ K^{-1}$. The bulk modulus in Te is $B = 19$ GPa.[25] It is known that electron temperature immediately after pump excitation of Te sample is extremely high due to excitation of hot carriers, which thermalizes to lattice temperature within a ps through electron-optic-phonon coupling.[26] The lattice temperature increases only few K given the low excitation density used in our low-temperature (10 K) experiment. Therefore, we can safely assume an upper bound increased lattice temperature of $\Delta T = 20$ K within 10 ps. Now, using all these parameters the photoinduced thermoelastic stress σ_{TH} can be estimated to be $|\sigma_{TH}| = 0.006$ GPa, which will be even smaller at later delay times. Next, the electron-acoustic deformation potential stress σ_{DP} can be estimated by:[19] $\sigma_{DP} = -a_{e-ac} \cdot \Delta N \approx -0.0003$ GPa, with $a_{e-ac} = -8.5$ eV along c-axis[27] and with photoinduced carrier density of $\Delta N \sim 2\times10^{23}$ m^{-3}. The stress perpendicular to c-axis increases to $\sigma_{DP} \approx 0.001$ GPa, with $a_{e-ac} = 35$ eV.[27] If we estimate the band gap change from these stress values,[28] these stress values are too small to induce tens of meV band gap shifts that we observed in our samples. Therefore, we argue that the piezoelectric strain induced by inverse piezoelectric effect (IPE) is the most likely mechanism of strain generation in our samples. Based on experimental observations, piezoelectric shear strain is dominant in our samples.

With the simple theoretical expression,[29, 30] $\Delta E_{split} \approx 4 \cdot \Psi_d \cdot \varepsilon_{xy}$, where ΔE_{split} is the band splitting or lifting, Ψ_d is the shear deformation potential and ε_{xy} is the shear strain, it is possible to estimate the shear strain value of the materials if we know the shear deformation potential and the resulting strain induced band splitting. The band splitting of $\Delta E_{split} = \Delta E_{lift} \sim 20$ meV is known from the TRS measurements at low temperature but the shear deformation potential is not known yet to the best of our knowledge. The typical value of deformation potential found in the literature is ~5 eV, which is estimated by the shift of energy gap in Te with dilation,[31] If we assume Ψ_d~5 eV in our case, the required shear strain turns out to be on the order of ~ 0.1 %, which is substantially lower than the value estimated by our ab initio DFT band structure calculations, i.e., $\sim 2-3$ %. Since the shear deformation potential is fundamentally different and complex, it is hard to predict the exact value of shear strain using this simple expression. However, considering required maximum shear strain of ~ 2 % to induce 20 meV band splitting, we can estimate the shear deformation potential in Te to be 0.25 eV. This simple estimation of photoinduced shear strain and shear deformation potential in Te will be very helpful for future studies since photoexcitation can be an alternative and non-destructive means for inducing exotic topological phases in Te.

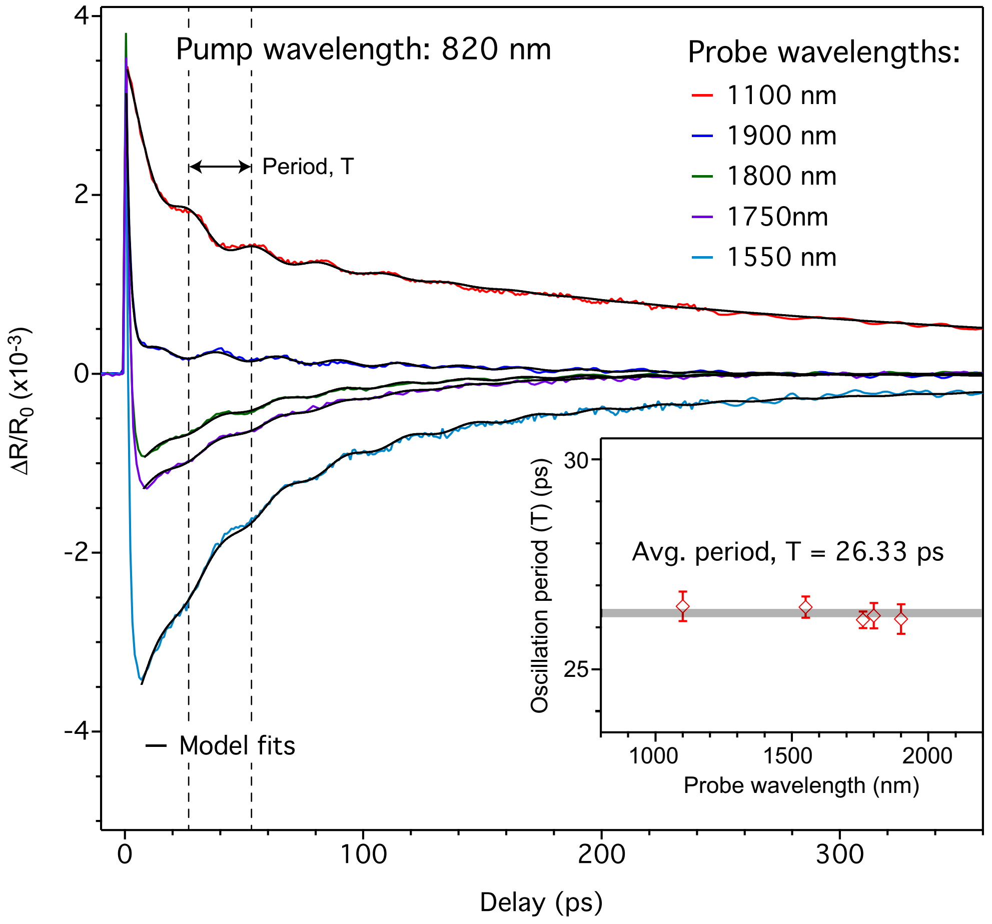

Supplementary Figure 8 | Coherent longitudinal acoustic phonon in Te nanosheets. Transient reflectance signal (TRS) of Te samples measured at different probe energies following 820 nm pump excitation at room temperature. Probe beam is polarized off-axis to ab-plane as well as c-axis but fixed for each wavelength. The TRS traces display weak oscillations embedded on a slowly decaying electronic signal. The oscillatory signal is caused by interference between the probe laser beam and photoinduced strain pulse propagation in the sample. Due to ultrathin samples, standing wave condition is fulfilled, which satisfy the linear dependence of oscillation period with film thickness, but does not depend on wavelength of the probe beam (see inset). This condition allows to estimate thickness knowing the acoustic wave velocity or vice versa through: $T = 2 \cdot d/v$, where d is sample thickness, T is the oscillation period, v is the velocity of longitudinal acoustic phonon, i.e., sound velocity, in Te. An average period of the oscillations is estimated to be 26.33 ps, which estimates the sound velocity of $\sim$ 1800 $\mathrm{m/s}$ in our 24 nm thick Te sample.

Supplementary References

1. Kresse, G. & Furthmuller, J. Efficient iterative schemes for ab initio total-energy calculations using a plane-wave basis set. *Phys. Rev. B* **54**, 11169 (1996).

2. Xue, X.-X.*, et al.* Strain tuning of electronic properties of various dimension elemental tellurium with broken screw symmetry. *J. Phys. Condens. Matter* **30**, 125001 (2018).

3. Agapito, L. A., Kioussis, N., Goddard, W. A. & Ong, N. P. Novel Family of Chiral-Based Topological Insulators: Elemental Tellurium under Strain. *Phys. Rev. Lett.* **110**, 176401 (2013).

4. Nakayama, K.*, et al.* Band splitting and Weyl nodes in trigonal tellurium studied by angle-resolved photoemission spectroscopy and density functional theory. *Phys. Rev. B* **95**, 125204 (2017).

5. Peng, H., Kioussis, N. & Snyder, G. J. Elemental tellurium as a chiral p-type thermoelectric material. *Phys. Rev. B* **89**, 195206 (2014).

6. Hirayama, M., Okugawa, R., Ishibashi, S., Murakami, S. & Miyake, T. Weyl Node and Spin Texture in Trigonal Tellurium and Selenium. *Phys. Rev. Lett.* **114**, 206401 (2015).

7. Aspnes, D. E. Third-derivative modulation spectroscopy with low-field electroreflectance. *Surf. Sci.* **37**, 418 (1973).

8. Tsirkin, S. S., Puente, P. A. & Souza, I. Gyrotropic effects in trigonal tellurium studied from first principles. *Phys. Rev. B* **97**, 035158 (2018).

9. Tutihasi, S., Roberts, G. G., Keezer, R. C. & Drews, R. E. Optical Properties of Tellurium in Fundamental Absorption Region. *Phy. Rev.* **177**, 1143 (1969).

10. Grosse, P. *Die Festkörpereigenschaften von Tellur*. Springer Berlin/Heidelberg (1969).

11. Loferski, J. J. Infrared Optical Properties of Single Crystals of Tellurium. *Phys. Rev.* **93**, 707 (1954).

12. Caldwell, R. S. & Fan, H. Y. Optical Properties of Tellurium and Selenium. *Phys. Rev.* **114**, 664 (1959).

13. Hardy, D. & Rigaux, C. Optical and Magnetooptical Studies of the Interband Transition of Holes in Tellurium. *phys. stat. sol.* **38**, 799 (1970).

14. Fiebig, T.*, et al.* Femtosecond dynamics of double proton transfer in a model DNA base pair: 7-azaindole dimers in the condensed phase. *J. Phys. Chem. A* **103**, 7419 (1999).

15. Lasher, G. & Stern, F. Spontaneous and Stimulated Recombination Radiation in Semiconductors. *Phys. Rev.* **133**, A553 (1964).

16. Bennett, B. R., Soref, R. A. & Del Alamo, J. A. Carrier-induced change in refractive index of InP, GaAs and InGaAsP. *IEEE J. Quantum Electron.* **26**, 113 (1990).

17. Hartig, P. A. & Loferski, J. J. Infrared Index of Refraction of Tellurium Crystals. *J. Opt. Soc. Am.* **44**, 17 (1954).

18. Thomsen, C., Strait, J., Vardeny, Z., Maris, H. J., Tauc, J. & Hauser, J. J. Coherent Phonon Generation and Detection by Picosecond Light Pulses. *Phys. Rev. Lett.* **53**, 989 (1984).

19. Thomsen, C., Grahn, H. T., Maris, H. J. & Tauc, J. Surface Generation and Detection of Phonons by Picosecond Light-Pulses. *Phys. Rev. B* **34**, 4129 (1986).

20. Ruello, P. & Gusev, V. E. Physical mechanisms of coherent acoustic phonons generation by ultrafast laser action. *Ultrasonics* **56**, 21 (2015).

21. Matsuda, O., Larciprete, M. C., Li Voti, R. & Wright, O. B. Fundamentals of picosecond laser ultrasonics. *Ultrasonics* **56**, 3 (2015).

22. Mante, P.-A., *et al.* THz acoustic phonon spectroscopy and nanoscopy by using piezoelectric semiconductor heterostructures. *Ultrasonics* **56**, 52 (2015).

23. Fukuda, S., Shiosaki, T. & Kawabata, A. Acousto-optic properties of tellurium at 10.6 μm. *J. Appl. Phys.* **50**, 3899 (1979).

24. Voloshinov, V. B., Balakshy, V. I., Kulakova, L. A. & Gupta, N. Acousto-optic properties of tellurium that are useful in anisotropic diffraction. *J. Opt. A: Pure Appl. Opt.* **10**, 095002 (2008).

25. Keller, R., Holzapfel, W. B. & Schulz, H. Effect of pressure on the atom positions in Se and Te. *Phys. Rev. B* **16**, 4404 (1977).

26. Hunsche, S., Wienecke, K., Dekorsy, T. & Kurz, H. Impulsive Softening of Coherent Phonons in Tellurium. *Phys. Rev. Lett.* **75**, 1815 (1995).

27. Enderlein, R. & Hache, A. Valence and Conduction Band Structure and Infrared Optical Properties of Tellurium in the Presence of Pressure. *phys. status solidi (b)* **60**, 739 (1973).

28. Anzin, V. B., Eremets, M. I., Kosichkin, Y. V., Nadezhdinskii, A. I. & Shirokov, A. M. Measurement of the energy gap in tellurium under pressure. *Phys. Status Solidi (a)* **42**, 385 (1977).

29. Hensel, J. C., Hasegawa, H. & Nakayama, M. Cyclotron Resonance in Uniaxially Stressed Silicon. II. Nature of Covalent Bond. *Phys. Rev.* **138**, A225 (1965).

30. Bir, G. L. & Pikus, G. E. *Symmetry and Strain-induced Effects in Semiconductors*. Wiley, New York (1974).

31. Bardeen, J. & Shockley, W. Deformation Potentials and Mobilities in Non-Polar Crystals. *Phys. Rev.* **80**, 72 (1950).
